# Measuring stigma affecting sex workers (SW) and men who have sex with men (MSM): A systematic review

**DOI:** 10.1371/journal.pone.0188393

**Published:** 2017-11-30

**Authors:** Alanna Fitzgerald-Husek, Michael J. Van Wert, Whitney F. Ewing, Ashley L. Grosso, Claire E. Holland, Rachel Katterl, Lori Rosman, Arnav Agarwal, Stefan D. Baral

**Affiliations:** 1 Dalla Lana School of Public Health, University of Toronto, Toronto, Ontario, Canada; 2 Community Psychiatry Program, Johns Hopkins Bayview Medical Center, Baltimore, Maryland, United States of America; 3 Centre for Public Health and Human Rights, Department of Epidemiology, Johns Hopkins Bloomberg School of Public Health, Johns Hopkins University, Baltimore, Maryland, United States of America; 4 HIV/AIDS, STIs & Viral Hepatitis Programme, World Health Organization Regional Office for Europe, Copenhagen, Denmark; 5 Welch Medical Library, Johns Hopkins School of Medicine, Johns Hopkins University, Baltimore, Maryland, United States of America; 6 School of Medicine, University of Toronto, Toronto, Ontario, Canada; 7 Department of Health Research Methods, Evidence, and Impact, McMaster University, Hamilton, Ontario, Canada; National and Kapodistrian University of Athens, GREECE

## Abstract

**Background:**

Stigma involves discrediting a person or group based on a perceived attribute, behaviour or reputation associated with them. Sex workers (SW) and men who have sex with men (MSM) are key populations who are often at increased risk for the acquisition and transmission of HIV and who are affected by stigma that can negatively impact their health and well-being. Although stigma was included as an indicator in the US National HIV/AIDS Strategic Plan and there have been consultations focused on adding a stigma indicator within PEPFAR and the Global Fund in relation to potentiating HIV risks among key populations, there remains limited consensus on the appropriate measurement of SW- or MSM-associated stigma. Consequently, this systematic review summarizes studies using quantitative, qualitative, or mixed methods approaches to measure stigma affecting sex workers and men who have sex with men.

**Methods and findings:**

This systematic review included English, French, and Spanish peer-reviewed research of any study design measuring SW- or MSM-associated stigma. Articles were published from January 1, 2004 to March 26, 2014 in PsycINFO, PubMed, EMBASE, CINAHL Plus, Global Health, and World Health Organization Global Health Library Regional Indexes.

Of the 541 articles reviewed, the majority measured stigma toward MSM (over 97%), were conducted in North America, used quantitative methods, and focused on internalized stigma.

**Conclusions:**

With the inclusion of addressing stigma in several domestic and international HIV strategies, there is a need to ensure the use of validated metrics for stigma. The field to date has completed limited measurement of stigma affecting sex workers, and limited measurement of stigma affecting MSM outside of higher income settings. Moving forward requires a concerted effort integrating validated metrics of stigma into health-related surveys and programs for key populations.

## Introduction

There is increasing interest and understanding of the adverse health outcomes associated with stigma [1]. Stigma involves marking and discrediting an individual or group on the basis of a real or perceived attribute, behaviour or membership to a group [1] and has been linked with negative outcomes at multiple levels. For individuals, studies have consistently found that stigma can result in lower self-esteem, poor academic achievement [2], and decreased uptake of health and social services [3]. At a social or macro level, stigma may influence legislation, policy decisions, insurance determinations, employment discrimination, and the orientation of research and theory [4, 5].

Of particular importance to those working in public health and health care policy and service delivery are the impacts of stigma on individuals’ mental and physical wellbeing. Among people living with HIV (PLHIV), studies show that higher stigma is associated with depression [6–8], anxiety [8], increased suicidality [9] and lower quality of life [10]. Higher stigma is also associated with a greater likelihood of chronic pain, poorer physical capacity [11], and morbidity related to lower levels of medication adherence [12–14]. Stigma may also influence health through mediators including lessened resourcefulness, negative effects on social relationships, and contributing to high stress levels for the affected individual [15].

People stigmatize others based on a series of social constructs, which vary across time and cultures [16]. Despite this, some groups, identities, and behaviours are consistently stigmatized across much of the world. Examples include stigma based on: sexual practices and identities of gay men and other men who have sex with men (MSM); occupationally-linked behaviours and identities of sex workers (SW); individuals who are transgender; substance use and addictions among people who use drugs; and health status of PLHIV [17–21]. Among these populations, a number of forms of stigma have been identified, including internalized, perceived, experienced, layered, and secondary stigmas [22]. Briefly, internalized stigma refers to a form of self-stigmatization whereby individuals accept negative judgments or attitudes applied to them [22]. Perceived or anticipated stigma is an awareness of devalued social status or expectation of discrimination based on a particular attribute [23]. Experienced or enacted stigma is the experience of a specific episode of discrimination against those with the stigmatized attribute or behaviour [24]. Secondary or courtesy stigma is stigma associated with those who have a connection with stigmatized individuals, such as their family or service providers [1, 25]. Layered or intersectional stigma [25] involves stigmas based on more than one attribute such as MSM living with HIV [25].

With growing recognition of the importance of stigma, there has been increasing interest and investment in stigma mitigation interventions [26]. Consequently, valid and reliable measures of stigma are needed to assess the impacts of these interventions and any changes in stigma over time [20]. Systematic reviews have examined measures of stigma affecting PLHIV [27]; less is known about measures of stigma affecting key populations whose sexual practices may put them at risk for HIV, including MSM and SW. An existing review on measuring attitudes towards homosexual men focused on stigma affecting a gay sexual orientation [28]. The systematic review presented here aims to summarize and synthesize studies that used quantitative, qualitative, or mixed methods to measure stigma affecting MSM and SW. Specifically, this review aims to systematically characterize how stigma associated with SW and with MSM is being measured and what validated and reliable stigma metrics exist for these key populations.

## Methods

### Search strategy

A scoping review of existing literature informed the development of this review’s search strategy and protocol (http://dx.doi.org/10.17504/protocols.io.ka6cshe). The search strategy used controlled vocabulary and subject headings, free text, and associated terms for both the stigma and key populations (SW and MSM) (Search strategies in S1 Text). Briefly, the Boolean operators “AND” and “OR” were used to combine the concepts. Cross-referencing concepts provided a broad, sensitive strategy to capture potentially relevant articles on SW- or MSM-associated stigma. Measurement-related terms were integrated in the abstract and full-text screening stages to identify relevant articles for inclusion, as including a measurement concept in the database search strategy created overly specific searches missing *a priori* determined key manuscripts. The base search was developed in the National Libraries of Medicine (PubMed) and adjusted according to other databases’ specifications. The following six databases were searched for peer-reviewed articles: PsycINFO, PubMed, EMBASE, CINAHL Plus, Global Health, and World Health Organization (WHO) Global Health Library Regional Indexes (AIM, LILACS, IMEMR, IMSEAR, and WPRIM).

### Eligibility criteria

#### Inclusion criteria

This review included primary research studies using quantitative, qualitative, and mixed methods data collection for the measurement of stigma associated with SW and/or MSM and published in English, French, or Spanish between January 1, 2004 and March 26, 2014. This timeframe of a decade was used to provide sufficient historical perspective on trends in stigma measurement. There were no restrictions on study design, duration or setting, country of study or publication, or on study population – including populations affected by stigma, perpetrators of stigma, students, healthcare workers – where stigma affecting SW or MSM was measured. Moreover, there were no restrictions on definitions, characteristics, identities or sexual practices of SW or MSM. For example, SW of any biological sex or gender identity, age, and race or ethnicity were included, and there were no limitations on definitions or types of sex work, nor on the duration or frequency of selling sex. MSM of any age, race or ethnicity were included, and there were no restrictions on type, duration, or frequency of same-sex sexual practices, including whether MSM had sex exclusively with other men, or also with women and/or transgender persons.

There were no limitations placed on the type, frequency, or duration of stigma associated with SW or MSM. The primary types of stigma of interest were decided through the scoping review and included internalized, perceived, and experienced stigmas, although studies including secondary/courtesy or other types of SW- or MSM-associated stigma were not excluded. Studies measuring MSM- or SW-associated stigma using pre-existing or new scales were included, regardless of whether validity or reliability were assessed, though levels of use of validated scales were noted.

#### Exclusion criteria

Studies measuring stigma without any form of a scale, with a single question, and/or using binary/dichotomous variables not combined into a scale (e.g. “Do you feel stigmatized?”) were excluded from final data abstraction. Dissertations and theses not published in peer-reviewed journals were excluded. To effectively study stigma affecting transgender populations, different and specific search strategies are required. Here, we did not exclude studies that included transgender people in larger studies with cisgender MSM though noted these when included, but we did not include transgender-specific stigma studies.

### Screening and abstraction

Independent reviewers were paired (e.g., reviewers 1 and 2; 1 and 3; 4 and 5) and each article was screened by two independent reviewers at the title and abstract (n = 6,470 entries) and full-text (n = 740 articles) review stages. Potentially relevant French and Spanish studies that had their titles and abstracts also translated into English were reviewed in the above manner and included in the above count. Otherwise, French and Spanish articles selected for full-text review – and those that subsequently met criteria for data abstraction – were completed by team members fluent in French or Spanish. Due to resource constraints, these few French and Spanish articles were completed by the single independent reviewer fluent in that language.

All English articles coded as potentially relevant by both reviewers were included for the next stage of the review process. If only one reviewer coded an article as potentially relevant during abstract screening, the review team included that entry for full-text review for increased sensitivity. After full-text review, discrepancies between reviewers regarding inclusion for data abstraction were resolved through discussions between the reviewers and another team member until consensus was reached.

Standardized forms were piloted and used for all screening phases and for data abstraction, per the search protocol. Data were abstracted by one reviewer for each included study using the developed standardized form, with a second reviewer independently examining 15% of articles and verifying their data abstraction. Independent dual abstraction of all included studies was not feasible due to resource constraints and the volume of included studies. The data abstraction form (S1 File) included information about study design and methods, study participants, target key population, and elements of stigma measurement, including scale, reliability, and validity. The form also included types of stigma (e.g. internalized, perceived, experienced); “N/A” was selected when the point of view was stigma perpetrators and the stigma types were not characterized.

## Results

**Fig 1:**
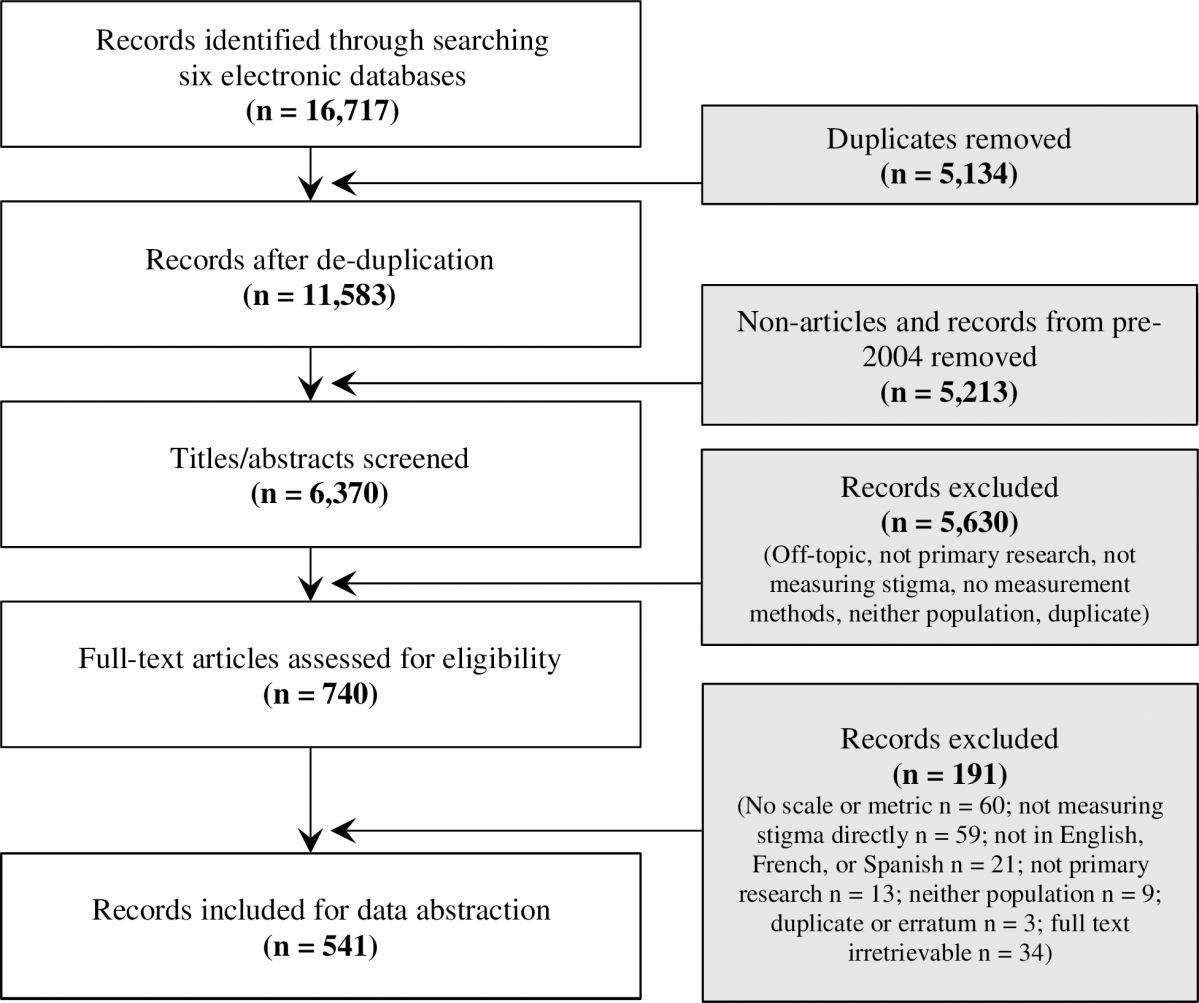
Flowchart of screening process for inclusion of articles measuring stigma affecting men who have sex with men (MSM) and stigma affecting sex workers (SW), 2004-2014.

The initial search strategy identified 16,717 entries between six electronic databases, of which 5,134 duplicates were removed and 5,213 were excluded as non-peer reviewed publications or those published before 2004. Titles and abstracts of the remaining 6,370 entries were screened: 5,630 (88.4%) were excluded based on eligibility criteria and 740 (11.6%) papers were eligible for full text review. Of these 740 articles, 199 (26.9%) were excluded and 541 (73.1%) articles were included in this review for data abstraction (S2 Text). Percent agreement between reviewer pairs ranged from 83% to 87% for abstract screening, and from 84% to 90% for full-text review. For details on the screening process, see the flowchart in Fig 1.

### Study characteristics

**Fig 2:**
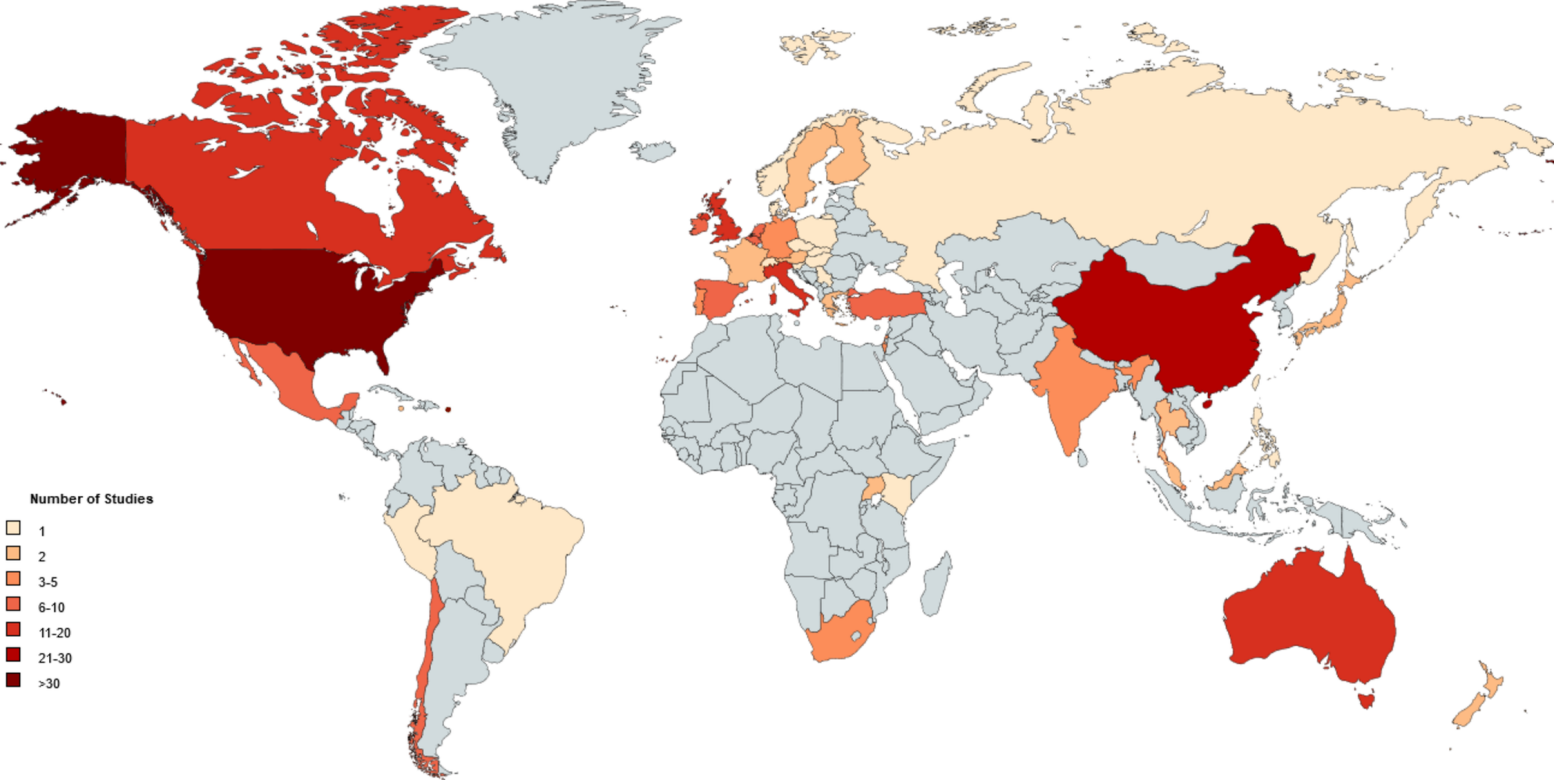
Geographic distribution of all included articles measuring stigma toward men who have sex with men (MSM) and stigma toward sex workers (SW) by country, 2004-2014. Not included in Fig 2 are studies that were Europe-wide [30, 31] and global without countries specified [32].

General characteristics of included studies are displayed in Table 1. Most articles (500/541; 92.4%) used quantitative methods, four (0.7%) used qualitative methods, and 37 (6.8%) used mixed methods. The majority of articles reported findings from North America: the United States (369/541; 68.2%) and Canada (19/541; 3.5%). Of the 369 articles from the United States, three (0.8%) measured SW stigma, one (0.3%) measured stigma for both SW and MSM, and the remaining 365 (98.9%) measured stigma for MSM populations. When grouped by WHO Regions [29], seven (1.3%) studies were from Sub-Saharan Africa (where HIV prevalence is highest) and measured MSM stigma. Seven (1.3%) articles reported findings from multiple regions. Location of data collection was unspecified in one (0.2%) article. Fig 2 provides the geographic distribution of included articles where data collection locations were specified, and Table 2 provides counts of each geographic location identified in included stigma measurement articles for SW only, SW and MSM, MSM only articles, as well as in papers identifying the inclusion of transgender persons.

**Table 1:** General characteristics of studies measuring stigma associated with men who have sex with men (MSM) and stigma associated with sex workers (SW) in articles from 2004-2014.

Characteristic		n (%)
Target Key Populations	**MSM cisgender or transgender populations**	**525 (97.0%)**
	Cisgender MSM populations	53 (10.1%)
	Transgender populations	10 (1.9%)
	Transgender and cisgender MSM populations	53 (10.1%)
	Unspecified whether only transgender or only cisgender MSM or both populations	409 (77.9%)
	**SW populations**	**13 (2.4%)**
	Female sex workers only	9 (69.2%)
	Gender not specified	4 (30.8%)
	**Both MSM and SW populations**	**3 (0.6%)**
	MSM + Female sex workers	1 (33.3%)
	Male sex workers only	1 (33.3%)
	MSM + SW genders not specified	1 (33.3%)
Methods	Quantitative	500 (92.4%)
	Qualitative	4 (0.7%)
	Mixed Methods	37 (6.8%)
Language of Publication	English	525 (97.0%)
	Spanish	12 (2.2%)
	French	2 (0.4%)
Publication Years	2004-2006	74 (13.7%)
	2007-2009	138 (25.5%)
	2010-2012	200 (37.0%)
	2013-2014	129 (23.8%)

**Table 2:** Distribution of identified geographic locations in included stigma measurement articles for sex workers (SW) only, SW and men who have sex with men (MSM), MSM only, and in papers including transgender persons, 2004-2014^a^.

Geographic locations identified	SW only papers	SW and MSM papers	MSM only papers	Transgender including papers (transgender only, and transgender and cisgender MSM)
Australia			19	1
Austria			2	
Barbados			1	
Belgium			9	2
Brazil			1	
Canada			19	3
Chile			6	1
China	4	1	11	1
Czech Republic			1	
Denmark			1	
Finland		1	1	
France			2	
Germany			4	
Greece			2	
Hong Kong (PRC)			5	2
Hungary			1	
India	1		3	
Ireland			6	1
Israel	1		3	
Italy			15	
Jamaica			2	
Japan	1		1	
Kenya			1	
Malaysia			2	1
Mexico	1		1	
Netherlands			7	
New Zealand			2	
Norway			1	
Peru			1	1
Philippines			1	1
Poland			1	
Portugal			4	1
Russia	1			
Serbia			1	
Singapore			3	1
Slovakia			1	
Slovenia			1	
South Africa			4	
Spain			9	1
Sweden			3	
Switzerland			1	
Taiwan			1	
Thailand	1		1	1
Turkey			9	
Uganda			2	
Ukraine			1	
United Kingdom			11	1
United States	3	1	365	51

^a^ Some studies identified more than one geographic location; articles that did not identify specific geographic locations are not included in Table 2.

Regarding study samples in the 541 articles, 118 (21.8%) articles’ study populations were MSM only, 182 (33.6%) studies’ populations were MSM and another population (not including SWs), seven (1.3%) studies’ populations were SW only, and one (0.2%) study population was SW and another population (not including MSM). Three (0.6%) articles’ study populations included both MSM and SW. Thirty-four (6.3%) articles did not detail study sample composition, and 196 (36.2%) explicitly specified study samples other than MSM or SW (e.g. university students, health care workers, teachers, community members).

### Stigma measure characteristics and metrics

#### Stigmatized populations addressed

The majority (525/541; 97.0%) of studies measured stigma affecting gay men and other MSM. Of these 525 papers, 53 (10.1%) measured MSM-associated stigma for cisgender males only, 53 (10.1%) MSM articles specified including transgender individuals in their focus, 10 (1.9%) focused on transgender populations and did not include cisgender MSM, and 409 (77.9%) did not specify whether they restricted to cisgender MSM (Table 1). Among 13 (2.4%) articles assessing SW-associated stigma, nine (69.2%) focused on female SW and four (30.8%) did not specify SW genders. In addition to the above counts, three (0.6%) articles assessed stigma associated with both MSM and SW populations. Among these three studies, one (33.3%) article focused on stigma toward female SW, one (33.3%) focused on male SW, and one (33.3%) did not specify SW genders; none of these three articles specified whether individuals were cisgender and/or transgender.

#### Types of stigma

Of the 541 studies, stigma type could be categorized for 281 (51.4%) articles. Of these 281, 102 (36.3%) articles measured two forms of stigma, and 26 (9.3%) had three stigma types. Of these 281 papers, 195 (69.4%) measured internalized stigma, 126 (44.8%) assessed experienced stigma, and 110 (39.1%) measured perceived stigma.

Among 525 articles measuring MSM stigma only, 273 articles (52%) could be categorized as one of the pre-determined types of stigma of interest (internalized, experienced/enacted, perceived/anticipated). Of these 273 articles, 193 (70.7%) measured internalized stigma, 125 (45.8%) measured experienced stigma, and 104 (38.1%) measured perceived stigma. Among 13 articles addressing SW stigma only, we were able to categorize seven (53.8%). Of those, five (71.4%) measured perceived stigma, one (14.3%) measured experienced stigma, and one (14.3%) measured internalized stigma. Among three articles addressing both MSM and SW stigmas, stigma type could be categorized for one (33.3%) paper, which measured both internalized and experienced stigma.

#### Stigma scales

**Fig 3:**
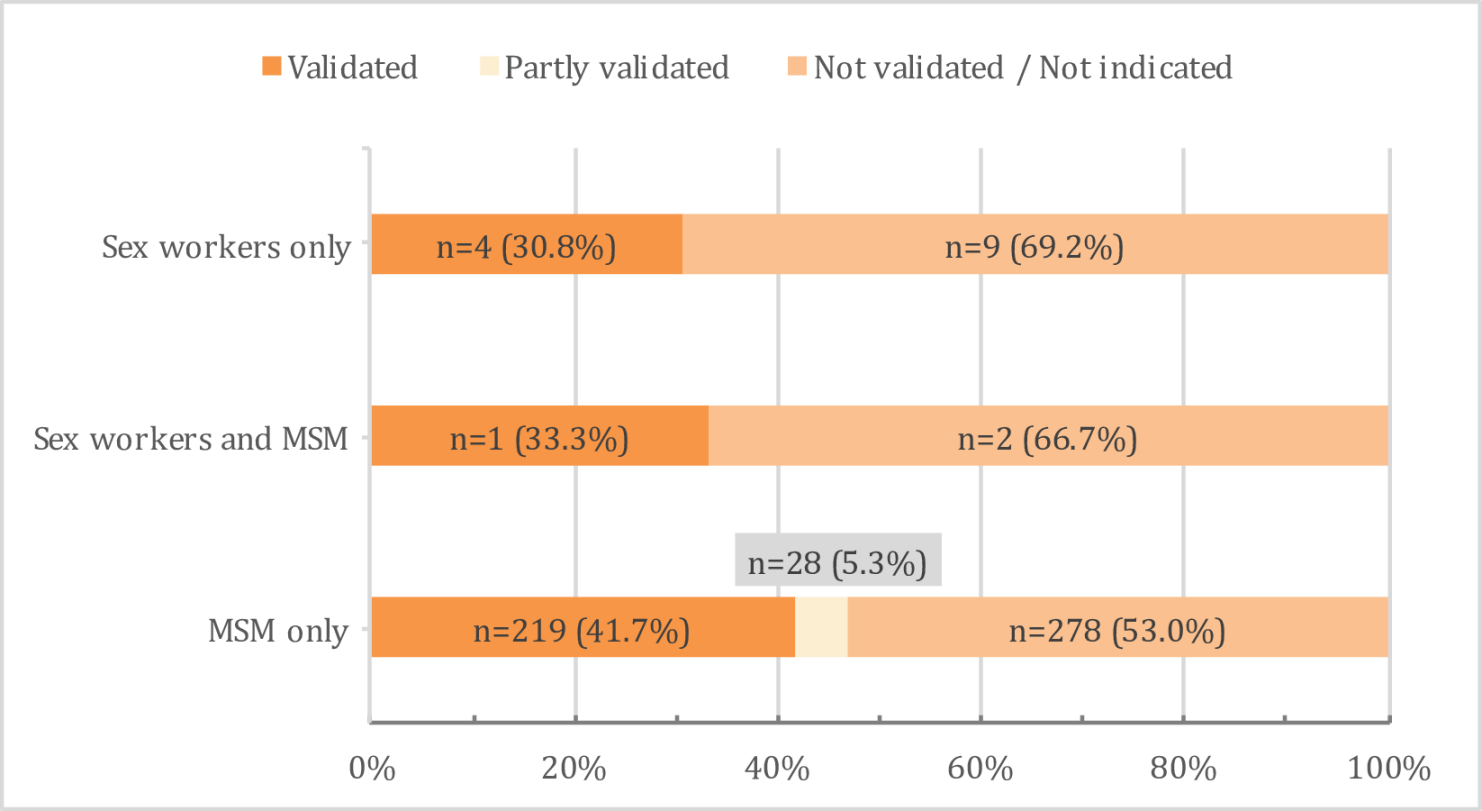
Percentage validated scales used to measure stigma associated with sex workers (SW), SW and men who have sex with men (MSM), and MSM in articles from 2004-2014.

Items from several stigma scales were commonly used and adapted. Some studies employed multiple stigma scales or components of different scales. Regarding reliability, 369 (68.2%) articles provided Cronbach’s alpha for the stigma scales used, while 79 (14.6%) articles referred to reliability of scales used but did not provide Cronbach’s alpha. Of the remaining 93 (17.2%) articles, 13 (2.4%) only reported reliability for some but not all stigma measures used, and 80 (14.8%) did not reference reliability. Fewer articles reported validity of measures used (Fig 3). Validated stigma measures were reported for 224 (41.4%) articles, 28 (5.2%) papers reported validation for some but not all stigma scales used, and 289 (53.4%) papers did not report on validity or did not use validated stigma metrics.

Among 525 articles addressing MSM stigma only, authors most commonly used items from the Attitudes Toward Lesbians and Gay Men (ATLG) Scale [33], which was used in 128 (24.4%) articles. Originally developed in 1984, the ATLG has been revised several times (e.g. 1987 [34], 1988 [35], 1993 [36], 1994 [37], 1997 [38], 1998 [39], 2004 [40]). The Reactions to Homosexuality Scale [41] was used in 19 (3.6%) articles. The Modern Homonegativity Scale (MHS) [42] and Experiences of Homophobia [43, 44] measures were each used in 18 (3.4%) articles. These and additional scales commonly used to assess MSM stigma are outlined in Table 3. Three hundred and sixty (68.6%) reported a Cronbach’s alpha for the MSM stigma scales used, 13 (2.4%) referenced reliability for some but not all scales used, and 75 (14.3%) did not reference reliability of stigma measures employed. Regarding validation, 220 (41.9%) articles reported validation of included stigma scales, 28 (5.3%) articles reported validation for some but not all measures used, and 277 (52.8%) did not report on validity or did not use validated metrics.

**Table 3:** Most commonly used scales measuring stigma associated with men who have sex with men (MSM) in articles from 2004-2014.

Author	Title	Years of Publication	Frequency of Use
Herek	Attitudes Toward Lesbians and Gay Men (ATLG)	1984 [33], 1987 [34], 1988 [35], 1993 [36], 1994 [37], 1997 [38], 1998 [39], 2004 [40]	128
Ross & Rosser	Reactions to Homosexuality Scale	1996 [41]	19
Morrison & Morrison	Modern Homonegativity Scale (MHS)	2002 [42]	18
Diaz et al.	Experiences of Homophobia	2001 [43], 2004 [44]	18
Shidlo	Revised Nungesser Homosexuality Attitudes Inventory (NHAI-R)	1994 [45]	16
Mayfield	Internalized Homonegativity Inventory (INHI)	2001 [46]	16
Hudson & Ricketts	Index of Homophobia (IHP)	1980 [47]	15
Martin & Dean	The Internalized Homophobia Scale (IHP)	1987 [48], 1992 [49]	13
Nungesser	Nungesser Homosexuality Attitudes Inventory (NHAI)	1983 [50]	12
Pinel	Stigma Consciousness Questionnaire (SCQ)	1999 [51]	2

Among 13 articles addressing SW stigma only, no single stigma scale was used more than once (Table 4). Of the 13 scales measuring SW stigma, six (46.2%) were created for the study in which they are referenced: Sex Worker Stigma Index [52]; Perceived Stigma of Purchasing Sex [53]; Attitudes Towards and Beliefs About Sex Work [53]; Perceived Stigma [54]; Self-perceived Stigma [55]; and Attitudes Toward Prostitutes and Prostitution Scale [56]. Of the 13 articles measuring SW-associated stigma, seven (53.8%) reported Cronbach’s alpha for the stigma scales used, one (7.7%) referred to reliability without providing Cronbach’s alpha, and five (38.5%) did not reference reliability of stigma measures used. Four (30.8%) articles reported validation of included stigma scales, and nine (69.2%) papers did not report on validity or did not use validated stigma metrics.

**Table 4:** Scales used to measure stigma associated with sex workers (SW) in articles from 2004-2014^a^.

Author	Scales	Years of Publication
Basow & Campanile	Attitudes Toward Prostitution Scale (ATP)	1990 [57]
Genberg et al.	HIV-Related Stigma Scale	2009 [58]
Harvey	Stigmatization Scale (short version)	2001 [59]
Held	General Attitudes Towards HIV and AIDS and People Who Are Infected	1993 [60]
Hong	Self-Perceived Stigma	2010 [55]
Jehu	Jehu Belief Inventory	1988 [61]
Kamise	Perceived Occupational Stigma (including subscales of the Stigma Awareness and Stereotype Threat Scale)	2010 [62]
Kelly et al.	The Social Interaction Scale (SIS)	1987 [63]
Kelly et al.	Prejudice Evaluation Scale (PES)	1987 [63]
Lau et al.	Dimensional Stigmatization Scale (DSS)	2007 [64]
Lau et al.	Overall Stigmatization Scales for Vulnerable Group (OSSVG)	2007 [64]
Levin	Attitudes toward Prostitutes and Prostitution Scale	2011 [56]
Liu et al.	Sex Worker Stigma Index	2011 [52]
Pinel	Stigma Consciousness Questionnaire (SCQ)	1999 [51]
Pitpitan	Attitudes Towards and Beliefs About Sex Work	2013 [53]
Pitpitan	Perceived Stigma of Purchasing Sex	2013 [53]
Zhang et al.	Perceived Stigma	2013 [54]

^a^Frequency of use is not listed as all scales measuring SW-related stigma were used only once.

Among the three articles addressing both MSM and SW stigma, no scale was used more than once. The Overall Stigmatization Scale for a Vulnerable Group (OSSVG) [64], Dimensional Stigmatization Scales [64], and the Stigma Consciousness Questionnaire [51] were each used once to measure both MSM- and SW-related stigmas. In one (33.3%) article, scales for MSM and SW-related stigmas were created using modified AIDS stigma scales [60]. Two (66.7%) articles reported Cronbach’s alpha for the stigma scales used, and one (33.3%) referenced reliability without providing Cronbach’s alpha. One (33.3%) article reported validation for included metrics and two (66.7%) did not report on validity or did not use validated scales.

## Discussion

This systematic review provides a comprehensive overview of how stigma affecting MSM and SW is being measured, with several relevant findings to inform future studies. Notably, while MSM and SW have been studied and characterized as being disproportionately affected by key health conditions globally, studies measuring MSM- and SW-associated stigmas have been conducted predominantly in high-income countries, with far fewer occurring in low- and middle-income settings, including Latin America, Sub-Saharan Africa, the Middle East, and North Africa. Separately, there is far less research measuring stigma affecting SW. Although there are a significant number of studies about MSM, these have traditionally focused on stigma related to sexual orientation or identity rather than sexual practices – which may limit utility in other settings given the cultural specificity of sexual orientation. Finally, there remains limited usage of validated indicators of stigma affecting these populations, suggesting that the field would greatly benefit from increased rigour in the measurement of stigma.

Ultimately, only 3.0% (16/541) of included studies specifically measured SW-associated stigma. Even fewer used existing validated stigma metrics developed for SW, which allow health workers to compare differences in stigma magnitude between individuals and groups, changes over time within individuals or groups, or differential effects of stigma reduction interventions. Some studies measured SW stigma by adapting existing stigma scales not specific to SW, such as The Social Interaction Scale [63, 65], The Prejudice Evaluation Scale [63], and The Stigmatization Scale (Short Scale) [59]. However, this review found a few scales created specifically for measuring SW-associated stigma, such as the Sex Worker Stigma Index [52], the Attitudes Towards Prostitutes and Prostitution Scale [56], the Sexual Network Questionnaire [66], and the cross-sectional scales developed by Zhang et al. (2013) [54] and Hong et al. (2010) [55] – where all scales but the latter two were reported as having been validated. Several potentially relevant studies identified through the search strategy were excluded in screening stages. While these studies acknowledged that stigma exists and may have identified public health imperatives or implemented public health interventions to address stigma for SW or empower SW to overcome stigmatizing attitudes, they did not actually operationally measure SW stigma (e.g. [67–69]). Moreover, some screened articles were excluded as they measured SW stigma in binary terms or with only one assessment question [70–72]. Overly simplified or single indicators limit the extent to which stigma can be quantified in a nuanced way. For the purposes of this review, we only included studies that explicitly measured SW-related stigma to understand the specific health needs of SW. However, given the diversity among SW – including male, female, and transgender SW – there is value in measuring the intersectionality of different stigmas affecting these populations [69], and to better understanding the potentially negative, synergistic effects of layered stigmas on SW [73].

The majority of studies examining MSM stigma have included a focus on sexual orientation constructs and the use of anti-gay/anti-homosexuality attitude scales [35, 41, 45], as Table 3 highlights. While these studies provide important information, they may be less sensitive in the measurement of stigma affecting men who have sex with other men but who do not self-identify as gay or homosexual [74], particularly in countries with different local terms and identities that do not fit easily into the MSM paradigm. Fewer studies have focused on measuring stigma associated with same-sex practices [74, 75]. Yet in some of the most stigmatizing environments [76–79], there is often independence of sexual orientation and sexual practices [80]. The criminalization of same-sex practices may also challenge stigma measurement and interventions due to potential danger and difficulty in undertaking these important endeavours [79], as has been noted recently in Uganda [81].

Around the globe, there may be different local terms, definitions and identities regarding which persons may self-identify as and/or be recognized under the umbrella term transgender. Results from this review indicated that there has been increased study of stigma and discrimination experienced by transgender persons. For example, the Transsexual Prejudice Scale [82] was used to examine interventions aiming to reduce transgender prejudice. A few transgender-specific stigma scales have been developed and validated, such as: the Transphobia Scale [83]; the Chinese Attitudes toward Transgenderism and Transgender Civil Rights Scale [84]; the cross-culturally validated [85] Genderism and Transphobia Scale [86]; and the Perceptions of the Averseness of Discrimination Scale (PADS) [87], which measures discrimination related to transgender status, and discrimination based on race/ethnicity. However, this review found some MSM studies tended to combine transgender women with MSM, or did not include subgroup analyses when both transgender women and cisgender MSM were included in the same study [88–90]. In a study outside the scope of this review, Bazargan & Galvan [91] adapted general scaled questions measuring perceived discrimination to evaluate transgender-specific maltreatment, assessing it among transgender women. Metrics that independently and specifically measure gender-related stigma among transgender populations are crucial, as is examining stigma associated with transgender women and with transgender men separately, given differential experiences and impacts of stigma between transgender men and women [92]. While there are some studies evaluating gender-related stigma experienced by transgender women [69, 91, 93, 94], there is a need for additional research measuring the intersectionality of gender-related, sexual practice-related, and HIV-related stigmas to further inform interventions.

Beyond the gaps identified above related to these key populations, this review highlighted the opportunity for increased standardization across settings in measurement and methodologically sound validation. Given heightened interest in the well-being of key populations around the world, there has been translation – and some validation – of stigma scales in different linguistic contexts, including Chinese [95–103], Spanish [85, 104–110], Turkish [111–115], Italian [106, 116–118], and Hebrew [119, 120]. Although increased stigma measurement across settings is an advance, there has been limited psychometric assessment of stigma metrics in many settings. Overall, the majority of included articles did not use validated stigma metrics. And while validation can take many forms – including content validity, face validity, and criterion-related validity – it represents an important component of ensuring appropriate measurement of stigma [121].

Content validation assesses whether a measure includes all important dimensions of stigma [121], and was done in certain studies, including one developing the Sexual Prejudice Scale [122]. Some included studies reported face validation of stigma metrics, relying on experts or members of affected populations to assess whether a scale’s items appeared to measure the right stigma concepts [122, 123]. Criterion-related validity can take different forms, including concurrent or construct validity. Concurrent validity is the extent a developed stigma metric corresponds with other established measures of the same concept [121]. Few included studies had multiple MSM- or SW-related stigma metrics compared with each other in the same questionnaire, though this is a common practice in studies of other types of stigma [124]. An exception was a study assessing the concurrent validity of the Multiple Discrimination Scale (MDS) by reporting its correlation with other instruments (e.g. the Internalized Sexual Orientation Stigma Measures [125]). Two types of construct validity are convergent and discriminant validity. Convergent validity – the extent a metric correlates with other related variables in the same datasets [121, 126] – was found for a scale assessing attitudes toward gay rights, as it correlated with gender and religion [121, 126]. Discriminant validity is the extent a metric is independent of other conceptually distinct measures in the same dataset [121]. For example, one included study measuring attitudes towards gay men measured and controlled for social desirability bias [127]. Another reviewed study demonstrated discriminant validity of the Internalized Homophobia Scale, reporting its lack of correlation with other distinct concepts: positive affect and hostility attitudes [115]. Reliability assesses a metric’s consistency [121] using internal consistency and test-retest methods. Most included studies assessed internal consistency by calculating or reporting Cronbach’s alpha of stigma measures. A test-retest approach to determine reliability (based on measuring the same concept twice [121]) was used in few studies: one demonstrated test-retest reliability of the Multiple Discrimination Scale through monthly administrations of the scale [125].

Some limitations of this review must be acknowledged, as well as the review’s strengths in synthesizing existing literature. The large volume of information reviewed and comprehensiveness of this review provided wide scope, but limited the ability to delve into specific details on particular aspects of stigma scale measurement. With the large volume of included articles, results were not dually abstracted for each study, although quality control was completed on 15% of extracted articles via independent review by a second reviewer. Any questions about a particular aspect of an article’s data abstraction were reviewed and discussed with other co-authors to maximize consensus and minimize subjectivity in the review process. Outside the comprehensive review of the six databases, no additional searches for unpublished or non-peer-reviewed sources were planned or undertaken, and hand searching of all 541 included articles’ reference lists was not conducted. However, including quantitative, qualitative and mixed methods research from six electronic databases and articles published in three widely-spoken languages helped minimize geographic, language, and publication bias, and represent strengths of this review. This large-scale review scoped and systematically characterized how and where SW- and MSM-associated stigma is being measured. Given this focus and the volume of included studies, critical appraisal was not done for each individual study included in this review. Additionally, while the methods used for this review were consistent with PRISMA guidelines, the protocol not was able to be registered in PROSPERO as the data collection had initiated before PROSPERO had emerged as the standard database in which to register systematic reviews. Finally, this review focused on cisgender MSM and did not appropriately include all of the terms for transgender men or women. If a study was focused on MSM without differentiating cisgender MSM from transgender women, then it was not excluded. A limitation of this review is that it did not adequately study approaches to measure transgender-related stigma which represents a key research question moving forward, especially given the intersectionality of sexual orientation, sexual practice, and gender-related stigma.

## Conclusion

The improved measurement of stigma has great potential in guiding effective responses to a variety of health conditions disproportionately affecting key populations. And while these data show significant measurement of MSM-related stigma, this work has primarily been completed in high-income settings. Moreover, where completed, there remains inconsistent use of validated stigma metrics. Moving forward necessitates improved measurement of stigma affecting SW as well as transgender persons, and also increased work for all populations – particularly across Sub-Saharan Africa and the Middle East. While not the focus here, there are limited data on stigma affecting people who use drugs, and these populations were not included in this review. The data in this review suggests the opportunity for the use of validated scales – or more efforts to validate scales – when measuring MSM- or SW-associated stigmas in new settings. Though contexts differ, key populations exist in every society around the world. There continue to be epidemiologic and interventional efforts to comprehensively characterize the specific HIV prevention, treatment, and care needs of these populations. The effective integration of stigma metrics into these studies and services will provide the opportunity to characterize the ideal content of biomedical and behavioural approaches to decrease proximal HIV acquisition and transmission risks, as well as optimal implementation strategies for mitigating the barriers to uptake of those services among those most in need.

## Supporting information

S1 Text Search strategies. (DOCX)

S2 Text Articles included for abstraction (N = 541). (DOCX)

S3 Text PRISMA checklist. (DOC)

S1 File Data abstraction form. (XLSX)

S2 File Data abstraction N = 541 articles. (XLSX)
