# Supplementary material for: Measuring stigma affecting sex workers (SW) and men who have sex with men (MSM): A systematic review
Source: PLoS One. 2017 Nov 30;12(11):e0188393. doi: 10.1371/journal.pone.0188393 (PMC5708696; doi:10.1371/journal.pone.0188393)
Supplement: S1 Text — (DOCX) [file pone.0188393.s001.docx]

**Search strategies**

PsycINFO Search Strategy:

| S1 | (DE "Homosexuality" OR DE "Male Homosexuality" OR DE "Same Sex Intercourse" OR DE "Transsexualism" OR DE "Transgender" OR DE "Transsexualism" OR DE "Bisexuality" OR DE "Prostitution") |
| --- | --- |
| S2 | TI ("MSM" OR "MSMW" OR "men who have sex with men" OR "sex with both" OR "male homosexual" OR "male homosexuals" OR "homosexual male" OR "homosexual males" OR "homosexual man" OR "homosexual men" OR "gay men" OR "gay man" OR "gay male" OR "gay males" OR homosexuality OR transgender* OR transsexual* OR "trans man" OR "trans men" OR transman OR transmen OR "trans woman" OR "trans women" OR transwoman OR transwomen OR "trans male" OR "trans males" OR "trans female" OR "trans females" OR bisexual* OR "LGBT" OR "GLBT" OR "sex work" OR "sex worker" OR "sex workers" OR prostitut* OR "transactional sex" OR "sex for money" OR "commercial sex" OR "call girl" OR "call girls") OR AB ("MSM" OR "MSMW" OR "men who have sex with men" OR "sex with both" OR "male homosexual" OR "male homosexuals" OR "homosexual male" OR "homosexual males" OR "homosexual man" OR "homosexual men" OR "gay men" OR "gay man" OR "gay male" OR "gay males" OR homosexuality OR transgender* OR transsexual* OR "trans man" OR "trans men" OR transman OR transmen OR "trans woman" OR "trans women" OR transwoman OR transwomen OR "trans male" OR "trans males" OR "trans female" OR "trans females" OR bisexual* OR "LGBT" OR "GLBT" OR "sex work" OR "sex worker" OR "sex workers" OR prostitut* OR "transactional sex" OR "sex for money" OR "commercial sex" OR "call girl" OR "call girls") |
| S3 | S1 OR S2 |
| S4 | (DE "Stigma" OR DE "Shame" OR DE "Prejudice" OR DE "Social Isolation" OR DE "Homosexuality (Attitudes Toward)" OR DE "Social Discrimination" OR DE "Social Perception" OR DE "Social Comparison" OR DE "Stereotyped Attitudes") |
| S5 | TI (shame OR stigma* OR stereotyp* OR prejudic* OR homonegativ* OR homoprejudice OR "social perception" OR "social perceptions" OR discriminat* OR alienation OR homophobi* OR antigay OR "anti-gay" OR "anti-homosexual" OR "anti-homosexuality" OR antihomosexual* OR "social isolation") OR AB (shame OR stigma* OR stereotyp* OR prejudic* OR homonegativ* OR homoprejudice OR "social perception" OR "social perceptions" OR discriminat* OR alienation OR homophobi* OR antigay OR "anti-gay" OR "anti-homosexual" OR "anti-homosexuality" OR antihomosexual* OR "social isolation") |
| S6 | S4 OR S5 |
| S7 | S3 AND S6 |

PubMed Search Strategy:

| #1 | "Homosexuality, Male"[Mesh] OR "Transsexualism"[Mesh] OR "Transgendered Persons"[Mesh] OR "Bisexuality"[Mesh] OR "Sex Workers"[Mesh] OR "Homosexuality"[Mesh:NoExp] |
| --- | --- |
| #2 | "MSM"[tw] OR "MSMW"[tw] OR "men who have sex with men"[tw] OR "sex with both"[tw] OR male homosexual*[tw] OR homosexual male*[tw] OR homosexual man[tw] OR homosexual men[tw] OR gay men[tw] OR gay man[tw] OR gay male*[tw] OR transgender*[tw] OR transsexual*[tw] OR trans man[tw] OR trans men[tw] OR transman[tw] OR transmen[tw] OR trans woman[tw] OR trans women[tw] OR transwoman[tw] OR transwomen[tw] OR trans male*[tw] OR trans female*[tw] OR bisexual*[tw] OR "LGBT"[tw] OR "GLBT"[tw] OR sex work*[tw] OR prostitut*[tw] OR transactional sex*[tw] OR "sex for money"[tw] OR commercial sex[tw] OR call girl*[tw] OR homosexuality[tiab] |
| #3 | #1 OR #2 |
| #4 | "Social Stigma"[Mesh] OR "Prejudice"[Mesh] OR "Shame"[Mesh] OR "Stereotyping"[Mesh] OR "Social perception"[Mesh] OR "Social Alienation"[Mesh] OR Homophobia[Mesh] OR "Social Discrimination"[Mesh] |
| #5 | shame[tiab] OR stigma*[tw] OR stereotyp*[tw] OR prejudic*[tw] OR homonegativ*[tw] OR homoprejudice[tw] OR social perception*[tw] OR discriminat*[tw] OR alienation[tw] OR social isolation[tw] OR homophobi*[tw] OR antigay[tw] OR "anti-gay"[tw] OR anti-homosexual*[tw] OR antihomosexual*[tw] |
| #6 | #4 OR #5 |
| #7 | #3 AND #6 |

EMBASE Search Strategy:

| #1 | ('male homosexuality'/exp OR 'homosexuality'/de OR 'men who have sex with men'/exp OR 'men who have sex with men and women'/exp OR 'transsexuality'/exp OR 'transgenderism'/exp OR 'male to female transsexual'/exp OR 'female to male transsexual'/exp OR 'LGBT people'/exp OR 'bisexuality'/exp OR 'bisexual male'/exp OR 'prostitution'/exp OR 'transactional sex'/exp) |
| --- | --- |
| #2 | ("MSM" OR "MSMW" OR "men who have sex with men" OR "sex with both" OR "male homosexual" OR "male homosexuals" OR "homosexual male" OR "homosexual males" OR "homosexual man" OR "homosexual men" OR "gay men" OR "gay man" OR "gay male" OR "gay males" OR homosexuality OR transgender* OR transsexual* OR "trans man" OR "trans men" OR transman OR transmen OR "trans woman" OR "trans women" OR transwoman OR transwomen OR "trans male" OR "trans males" OR "trans female" OR "trans females" OR bisexual* OR "LGBT" OR "GLBT" OR "sex work" OR "sex worker" OR "sex workers" OR prostitut* OR "transactional sex" OR "sex for money" OR "commercial sex" OR "call girl" OR "call girls"):ti,ab |
| #3 | #1 OR #2 |
| #4 | ('social stigma'/exp OR 'stigma'/exp OR 'prejudice'/exp OR 'shame'/exp OR 'social isolation'/exp OR 'homophobia'/exp OR 'social discrimination'/exp) |
| #5 | (shame OR stigma* OR stereotyp* OR prejudic* OR homonegativ* OR homoprejudice OR social perception* OR discriminat* OR alienation OR homophobi* OR antigay OR "anti-gay" OR (anti NEXT/1 homosexual*) OR antihomosexual* OR "social isolation"):ti,ab |
| #6 | #4 OR #5 |
| #7 | #3 AND #6 |

CINAHL Plus Search Strategy:

| S1 | (MH "Homosexuals, Male") OR (MH "Homosexuals") OR (MH "Homosexuality") OR (MH "Bisexuality") OR (MH "Bisexuals") OR (MH "GLBT Persons") OR (MH "Transsexualism") OR (MH "Transsexuals") OR (MH "Transgendered Persons") OR (MH "Prostitution") |
| --- | --- |
| S2 | TI ("MSM" OR "MSMW" OR "men who have sex with men" OR "sex with both" OR "male homosexual" OR "male homosexuals" OR "homosexual male" OR "homosexual males" OR "homosexual man" OR "homosexual men" OR "gay men" OR "gay man" OR "gay male" OR "gay males" OR homosexuality OR transgender* OR transsexual* OR "trans man" OR "trans men" OR transman OR transmen OR "trans woman" OR "trans women" OR transwoman OR transwomen OR "trans male" OR "trans males" OR "trans female" OR "trans females" OR bisexual* OR "LGBT" OR "GLBT" OR "sex work" OR "sex worker" OR "sex workers" OR prostitut* OR "transactional sex" OR "sex for money" OR "commercial sex" OR "call girl" OR "call girls") OR AB ("MSM" OR "MSMW" OR "men who have sex with men" OR "sex with both" OR "male homosexual" OR "male homosexuals" OR "homosexual male" OR "homosexual males" OR "homosexual man" OR "homosexual men" OR "gay men" OR "gay man" OR "gay male" OR "gay males" OR homosexuality OR transgender* OR transsexual* OR "trans man" OR "trans men" OR transman OR transmen OR "trans woman" OR "trans women" OR transwoman OR transwomen OR "trans male" OR "trans males" OR "trans female" OR "trans females" OR bisexual* OR "LGBT" OR "GLBT" OR "sex work" OR "sex worker" OR "sex workers" OR prostitut* OR "transactional sex" OR "sex for money" OR "commercial sex" OR "call girl" OR "call girls") |
| S3 | S1 OR S2 |
| S4 | (MH "Stigma") OR (MH "Prejudice") OR (MH "Shame") OR (MH "Social Isolation") OR (MH "Homophobia") OR (MH "Social Alienation") |
| S5 | TI (shame OR stigma* OR stereotyp* OR prejudic* OR homonegativ* OR homoprejudice OR "social perception" OR "social perceptions" OR discriminat* OR alienation OR homophobi* OR antigay OR "anti-gay" OR "anti-homosexual" OR "anti-homosexuality" OR antihomosexual* OR "social isolation") OR AB (shame OR stigma* OR stereotyp* OR prejudic* OR homonegativ* OR homoprejudice OR "social perception" OR "social perceptions" OR discriminat* OR alienation OR homophobi* OR antigay OR "anti-gay" OR "anti-homosexual" OR "anti-homosexuality" OR antihomosexual* OR "social isolation") |
| S6 | S4 OR S5 |
| S7 | S3 AND S6 |

Global Health Search Strategy:

| 1. | (exp homosexuality/ OR exp men who have sex with men/ OR exp bisexuality/ OR exp prostitution/ OR exp prostitutes/ OR exp sex workers/) |
| --- | --- |
| 2. | (MSM OR MSMW OR men who have sex with men OR sex with both OR male homosexual* OR homosexual male* OR homosexual man OR homosexual men OR homosexuality OR gay men OR gay man OR gay male* OR transgender* OR transsexual* OR trans man OR trans men OR transman OR transmen OR trans woman OR trans women OR transwoman OR transwomen OR trans male* OR trans female* OR bisexual* OR LGBT OR GLBT OR sex work* OR prostitut* OR transactional sex* OR "sex for money" OR commercial sex OR call girl*).tw. |
| 3. | 1 OR 2 |
| 4. | (exp stigma/ or exp social stigma/) |
| 5. | (shame OR stigma* OR stereotyp* OR prejudic* OR homonegativ* OR homoprejudice OR social perception* OR discriminat* OR alienation OR homophobi* OR antigay OR anti-gay OR anti homosexual* OR antihomosexual* OR "social isolation").tw. |
| 6. | 4 OR 5 |
| 7. | 3 AND 6 |

WHO Global Health Library Search Strategy:

| (MSM OR MSMW OR men who have sex with men OR homosexual OR gay OR transgender$ OR transsexual$ OR bisexual$ OR LGBT OR GLBT OR sex work$ OR prostitute$ OR transactional sex$ OR sex for money OR commercial sex OR call girl$ OR homosexuality) AND (shame OR stigma$ OR stereotyp$ OR prejudice$ OR homonegativ$ OR homoprejudice OR social perception$ OR discriminat$ OR alienation OR social isolation OR homophobi$ OR antigay OR anti homosexual OR antihomosexual$) |
| --- |
