# Supplementary material for: Measuring stigma affecting sex workers (SW) and men who have sex with men (MSM): A systematic review
Source: PLoS One. 2017 Nov 30;12(11):e0188393. doi: 10.1371/journal.pone.0188393 (PMC5708696; doi:10.1371/journal.pone.0188393)
Supplement: S2 Text — (DOCX) [file pone.0188393.s002.docx]

**Articles included for abstraction (N = 541)**

1. Adolfsen A, Iedema J, Keuzenkamp S. Multiple dimensions of attitudes about homosexuality: development of a multifaceted scale measuring attitudes toward homosexuality. J Homosex 2010;57(10):1237-57.

2. Adrien A, Beaulieu M, Leaune V, Perron M, Dassa C. Trends in attitudes toward people living with HIV, homophobia, and HIV transmission knowledge in Quebec, Canada (1996, 2002, and 2010). AIDS Care 2013;25(1):55-65.

3. Aerts S, Van Houtte M, Dewaele A, Cox N, Vincke J. Sense of belonging in secondary schools: a survey of LGB and heterosexual students in Flanders. J Homosex 2012;59(1):90-113.

4. Ahrold TK, Meston CM. Ethnic differences in sexual attitudes of U.S. college students: Gender, acculturation, and religiosity factors. Archives of Sexual Behavior 2010;39(1):190-202.

5. Alderson KG, Orzeck TL, McEwen SC. Alberta high school counsellors’ knowledge of homosexuality and their attitudes toward gay males. Canadian Journal of Education 2009;32(1):87-117.

6. Amadio DM. Internalized heterosexism, alcohol use, and alcohol-related problems among lesbians and gay men. Addict Behav 2006;31(7):1153-62.

7. Amadio DM, Chung YB. Internalized Homophobia and Substance Use Among Lesbian, Gay, and Bisexual Persons. Journal of Gay & Lesbian Social Services: Issues in Practice, Policy & Research 2004;17(1):83-101.

8. Aosved AC, Long PJ. Co-occurrence of rape myth acceptance, sexism, racism, homophobia, ageism, classism, and religious intolerance. Sex Roles 2006;55(7-8):481-492.

9. Arndt M, de Bruin K. Measurement of attitudes toward bisexual men and women among South African university students: the validation of an instrument. J Homosex 2011;58(4):497-520.

10. Arnold O, Voracek M, Musalek M, Springer-Kremser M. Austrian medical students' attitudes towards male and female homosexuality: a comparative survey. Wien Klin Wochenschr 2004;116(21-22):730-6.

11. Averett PE, Hegde A. School social work and early childhood student's attitudes toward gay and lesbian families. Teaching in Higher Education 2012;17(5):537-549.

12. Ayala G, Bingham T, Kim J, Wheeler DP, Millett GA. Modeling the impact of social discrimination and financial hardship on the sexual risk of HIV among Latino and Black men who have sex with men. Am J Public Health 2012;102 Suppl 2:S242-9.

13. Baams L, Beek T, Hille H, Zevenbergen FC, Bos HM. Gender nonconformity, perceived stigmatization, and psychological well-being in Dutch sexual minority youth and young adults: a mediation analysis. Arch Sex Behav 2013;42(5):765-73.

14. Baiocco R, Argalia M, Laghi F. The Desire to Marry and Attitudes Toward Same-Sex Family Legalization in a Sample of Italian Lesbians and Gay Men. Journal of Family Issues 2014;35(2):181-200.

15. Baiocco R, D'Alessio M, Laghi F. Binge drinking among gay, and lesbian youths: The role of internalized sexual stigma, self-disclosure, and individuals' sense of connectedness to the gay community. Addict Behav 2010;35(10):896-9.

16. Baiocco R, Fontanesi L, Santamaria F, Ioverno S, Baumgartner E, Laghi F. Coming out during adolescence: Perceived parents' reactions and internalized sexual stigma. Journal of Health Psychology 2014.

17. Baiocco R, Ioverno S, Cerutti R, Santamaria F, Fontanesi L, Lingiardi V, et al. Suicidal ideation in Spanish and Italian lesbian and gay young adults: The role of internalized sexual stigma. Psicothema 2014;26(4):490-496.

18. Baiocco R, Laghi F, Di Pomponio I, Nigito CS. Self-disclosure to the best friend: friendship quality and internalized sexual stigma in Italian lesbian and gay adolescents. J Adolesc 2012;35(2):381-7.

19. Baiocco R, Nardelli N, Pezzuti L, Lingiardi V. Attitudes of italian heterosexual older adults towards lesbian and gay parenting. Sexuality Research & Social Policy: A Journal of the NSRC 2013.

20. Baker LM, Wilson FL, Winebarger A. An exploratory study of the health problems, stigmatization, life satisfaction, and literacy skills of urban, street-level sex workers. Women Health 2004;39(2):83-96.

21. Balsam KF, Beadnell B, Molina Y. The Daily Heterosexist Experiences Questionnaire: Measuring Minority Stress Among Lesbian, Gay, Bisexual, and Transgender Adults. Measurement & Evaluation in Counseling & Development (Sage Publications Inc. ) 2013;46(1):3-25.

22. Balsam KF, Mohr JJ. Adaptation to Sexual Orientation Stigma: A Comparison of Bisexual and Lesbian/Gay Adults. J Couns Psychol 2007;54(3):306-319.

23. Balsam KF, Molina Y, Beadnell B, Simoni J, Walters K. Measuring multiple minority stress: the LGBT People of Color Microaggressions Scale. Cultur Divers Ethnic Minor Psychol 2011;17(2):163-74.

24. Barbosa P, Torres H, Silva MA, Khan N. Agapé Christian reconciliation conversations: Exploring the intersections of culture, religiousness, and homosexual identity in Latino and European Americans. Journal of Homosexuality 2010;57(1):98-116.

25. Barnes DM, Meyer IH. Religious affiliation, internalized homophobia, and mental health in lesbians, gay men, and bisexuals. Am J Orthopsychiatry 2012;82(4):505-15.

26. Barrientos J, Cárdenas M, Gómez F, Frías-Navarro D. Assessing the dimensionality of Beliefs About Children's Adjustment in Same-Sex Families Scale (BCASSFS) in Chile. Sexuality Research & Social Policy: A Journal of the NSRC 2013;10(1):43-51.

27. Barron JM, Struckman-Johnson C, Quevillon R, Banka SR. Heterosexual men's attitudes toward gay men: A hierarchical model including masculinity, openness, and theoretical explanations. Psychology of Men & Masculinity 2008;9(3):154-166.

28. Bassett RL, Kirnan R, Hill M, Schultz A. SOAP: Validating the Sexual Orientation and Practice Scale. Journal of Psychology and Christianity 2005;24(2):165-175.

29. Bassett RL, Van Nikkelen-Kuyper M, Johnson D, Miller A, Carter A, Grimm JP. Being a good neighbor: Can students come to value homosexual persons? Journal of Psychology and Theology 2005;33(1):17-26.

30. Bauermeister JA, Johns MM, Sandfort TG, Eisenberg A, Grossman AH, D'Augelli AR. Relationship trajectories and psychological well-being among sexual minority youth. J Youth Adolesc 2010;39(10):1148-63.

31. Bauermeister JA, Morales M, Seda G, Gonzalez-Rivera M. Sexual prejudice among Puerto Rican young adults. J Homosex 2007;53(4):135-61.

32. Baunach DM, Burgess EO. HIV/AIDS prejudice in the American deep south. Sociological Spectrum 2013;33(2):175-195.

33. Baunach DM, Burgess EO, Muse CS. Southern (dis)comfort: Sexual prejudice and contact with gay men and lesbians in the south. Sociological Spectrum 2010;30(1):30-64.

34. Berg RC, Ross MW, Weatherburn P, Schmidt AJ. Structural and environmental factors are associated with internalised homonegativity in men who have sex with men: findings from the European MSM Internet Survey (EMIS) in 38 countries. Soc Sci Med 2013;78:61-9.

35. Berghe WV, Dewaele A, Cox N, Vincke J. Minority-specific determinants of mental well-being among lesbian, gay, and bisexual youth. Journal of Applied Social Psychology 2010;40(1):153-166.

36. Bernstein M, Swartwout P. Gay officers in their midst: heterosexual police employees' anticipation of the consequences for coworkers who come out. J Homosex 2012;59(8):1145-66.

37. Bhaskar R, B. U A, Komala M. A study on sexual attitudes of youth in Mysore Taluka. Journal of Psychosocial Research 2012;7(1):101-110.

38. Bingham TA, Harawa NT, Williams JK. Gender role conflict among African American men who have sex with men and women: associations with mental health and sexual risk and disclosure behaviors. Am J Public Health 2013;103(1):127-33.

39. Birkett M, Espelage DL, Koenig B. LGB and questioning students in schools: the moderating effects of homophobic bullying and school climate on negative outcomes. J Youth Adolesc 2009;38(7):989-1000.

40. Blackwell CW. Belief in the "free choice" model of homosexuality: a correlate of homophobia in registered nurses. J LGBT Health Res 2007;3(3):31-40.

41. Blackwell CW, Kiehl EM. Homophobia in registered nurses: impact on LGB youth. Journal of LGBT Youth 2008;5(4):28-48.

42. Bockting WO, Miner MH, Swinburne Romine RE, Hamilton A, Coleman E. Stigma, mental health, and resilience in an online sample of the US transgender population. Am J Public Health 2013;103(5):943-51.

43. Bogart L, Wagner G, Galvan F, Klein D, Lee M. Longitudinal relationships of HIV-, Race-, and sexual orientation-related discrimination to medication adherence among African-American men with HIV. J Int Assoc Physicians AIDS Care 2010;9(4):241.

44. Bogart LM, Landrine H, Galvan FH, Wagner GJ, Klein DJ. Perceived discrimination and physical health among HIV-positive Black and Latino men who have sex with men. AIDS Behav 2013;17(4):1431-41.

45. Boone TL, Duran A. Sexual prejudice among heterosexual college men as a predictor of condom attitudes. Sex Roles 2009;61(3-4):167-177.

46. Bos HMW, Picavet C, Sandfort TGM. Ethnicity, gender socialization, and children’s attitudes toward gay men and lesbian women. Journal of Cross-Cultural Psychology 2012;43(7):1082-1094.

47. Bosetti GL, Voci A, Pagotto L. Religiosity, the sinner, and the sin: Different patterns of prejudice toward homosexuals and homosexuality. TPM-Testing, Psychometrics, Methodology in Applied Psychology 2011;18(3):157-170.

48. Boysen GA. Biological explanations and stigmatizing attitudes: using essentialism and perceived dangerousness to predict antistigma intervention effectiveness. J Soc Psychol 2011;151(3):274-91.

49. Breen AB, Karpinski A. Implicit and explicit attitudes toward gay males and lesbians among heterosexual males and females. The Journal of Social Psychology 2013;153(3):351-374.

50. Brennan DJ, Craig SL, Thompson DE. Factors associated with a drive for muscularity among gay and bisexual men. Cult Health Sex 2012;14(1):1-15.

51. Brennan DJ, Crath R, Hart TA, Gadalla T, Gillis L. Body dissatisfaction and disordered eating among men who have sex with men in Canada. International Journal of Men's Health 2011;10(3):253-268.

52. Brenner BR, Lyons HZ, Fassinger RE. Can heterosexism harm organizations? Predicting the perceived organizational citizenship behaviors of gay and lesbian employees. The Career Development Quarterly 2010;58(4):321-335.

53. Brewster ME, Moradi B. Perceived experiences of anti-bisexual prejudice: instrument development and evaluation. Journal of Counseling Psychology 2010;57(4):451-468.

54. Brewster ME, Moradi B, Deblaere C, Velez BL. Navigating the borderlands: the roles of minority stressors, bicultural self-efficacy, and cognitive flexibility in the mental health of bisexual individuals. J Couns Psychol 2013;60(4):543-56.

55. Brewster ME, Velez B, DeBlaere C, Moradi B. Transgender individuals' workplace experiences: the applicability of sexual minority measures and models. J Couns Psychol 2012;59(1):60-70.

56. Brown J, Trevethan R. Shame, internalized homophobia, identity formation, attachment style, and the connection to relationship status in gay men. Am J Mens Health 2010;4(3):267-76.

57. Brown MJ, Groscup JL. Homophobia and acceptance of stereotypes about gays and lesbians. Individual Differences Research 2009;7(3):159-167.

58. Brown MJ, Henriquez E. Support for gay and lesbian civil rights: Development and examination of a new scale. Journal of Homosexuality 2011;58(4):462-475.

59. Brown TL, Alderson KG. Sexual identity and heterosexual male students' usage of homosexual insults: An exploratory study. Canadian Journal of Human Sexuality 2010;19(1-2):27-42.

60. Buck DM, Plant EA, Ratcliff J, Zielaskowski K, Boerner P. Concern over the misidentification of sexual orientation: social contagion and the avoidance of sexual minorities. J Pers Soc Psychol 2013;105(6):941-60.

61. Burns MN, Kamen C, Lehman KA, Beach SR. Attributions for discriminatory events and satisfaction with social support in gay men. Arch Sex Behav 2012;41(3):659-71.

62. Burns MN, Kamen C, Lehman KA, Beach SRH. Minority stress and attributions for discriminatory events predict social anxiety in gay men. Cognitive Therapy and Research 2012;36(1):25-35.

63. Bush A, Anderson E, Carr S. The declining existence of men's homophobia in British sport. Journal for the Study of Sports and Athletes in Education 2012;6(1):107-120.

64. Butler B. Death qualification and prejudice: the effect of implicit racism, sexism, and homophobia on capital defendants' right to due process. Behav Sci Law 2007;25(6):857-67.

65. Callahan MP, Vescio TK. Core American values and the structure of antigay prejudice. J Homosex 2011;58(2):248-62.

66. Calzo JP, Ward LM. Media exposure and viewers’ attitudes toward homosexuality: Evidence for mainstreaming or resonance? Journal of Broadcasting & Electronic Media 2009;53(2):280-299.

67. Camilleri P, Ryan M. Social Work Students' Attitudes toward Homosexuality and Their Knowledge and Attitudes toward Homosexual Parenting as an Alternative Family Unit: An Australian Study. Social Work Education 2006;25(3):288-304.

68. Cárdenas M, Barrientos J. Actitudes explícitas e implícitas hacia los hombres homosexuales en una muestra de estudiantes universitarios en Chile. Psykhe: Revista de la Escuela de Psicología 2008;17(2):17-25.

69. Cárdenas M, Barrientos J, Gómez F, Frías-Navarro D. Attitudes Toward Gay Men and Lesbians and Their Relationship with Gender Role Beliefs in a Sample of Chilean University Students. International Journal of Sexual Health 2012;24(3):226-236.

70. Cárdenas M, Barrientos JE. The attitudes toward lesbians and gay men scale (ATLG): adaptation and testing the reliability and validity in Chile. J Sex Res 2008;45(2):140-9.

71. Carrera-Fernández MV, Lameiras-Fernández M, Rodriguez-Castro Y, Vallejo-Medina P. Bullying among Spanish secondary education students: the role of gender traits, sexism, and homophobia. J Interpers Violence 2013;28(14):2915-40.

72. Carrera-Fernández MV, Lameiras-Fernández M, Rodriguez-Castro Y, Vallejo-Medina P. Spanish Adolescents' Attitudes toward Transpeople: Proposal and Validation of a Short Form of the Genderism and Transphobia Scale. The Journal of Sex Research 2014;51(6):654-666.

73. Carroll L, Güss D, Hutchinson KS, Gauler AA. How do U.S. students perceive trans persons? Sex Roles 2012;67(9-10):516-527.

74. Carter LW, Mollen D, Smith NG. Locus of control, minority stress, and psychological distress among lesbian, gay, and bisexual individuals. J Couns Psychol 2014;61(1):169-75.

75. Carvalho A, Lewis R, Derlega V, Winstead B, Viggiano C. Internalized Sexual Minority Stressors and Same-Sex Intimate Partner Violence. Journal of Family Violence 2011;26(7):501-509.

76. Case KA, Stewart B. Changes in diversity course student prejudice and attitudes toward heterosexual privilege and gay marriage. Teaching of Psychology 2010;37(3):172-177.

77. Case KA, Stewart B. Intervention Effectiveness in Reducing Prejudice Against Transsexuals. Journal of LGBT Youth 2013;10(1/2):140-158.

78. Chan KY, Reidpath DD. Stigmatization of patients with AIDS: understanding the interrelationships between Thai nurses' attitudes toward HIV/AIDS, drug use, and commercial sex. AIDS Patient Care STDS 2007;21(10):763-75.

79. Chan KY, Yang Y, Li ZR, Stoove MA, Reidpath DD. Interrelationships between HIV/AIDS and risk behavior prejudice among medical students in Southern China. Curr HIV Res 2009;7(6):601-11.

80. Chan KY, Yang Y, Zhang KL, Reidpath DD. Disentangling the stigma of HIV/AIDS from the stigmas of drugs use, commercial sex and commercial blood donation - a factorial survey of medical students in China. BMC Public Health 2007;7:280.

81. Chapman R, Watkins R, Zappia T, Nicol P, Shields L. Nursing and medical students' attitude, knowledge and beliefs regarding lesbian, gay, bisexual and transgender parents seeking health care for their children. J Clin Nurs 2012;21(7-8):938-45.

82. Chen YC, Tryon GS. Dual minority stress and Asian American gay men's psychological distress. Journal of Community Psychology 2012;40(5):539-554.

83. Choi KH, Hudes ES, Steward WT. Social discrimination, concurrent sexual partnerships, and HIV risk among men who have sex with men in Shanghai, China. AIDS Behav 2008;12(4 Suppl):S71-7.

84. Choi KH, Paul J, Ayala G, Boylan R, Gregorich SE. Experiences of discrimination and their impact on the mental health among African American, Asian and Pacific Islander, and Latino men who have sex with men. Am J Public Health 2013;103(5):868-74.

85. Chonody J, Woodford MR, Smith S, Silverschanz P. Christian Social Work Students' Attitudes Toward Lesbians and Gay Men: Religious Teachings, Religiosity, and Contact. Journal of Religion & Spirituality in Social Work 2013;32(3):211-226.

86. Chonody JM. Measuring sexual prejudice against gay men and lesbian women: development of the Sexual Prejudice Scale (SPS). J Homosex 2013;60(6):895-926.

87. Chonody JM, Rutledge SE, Smith S. 'That's so gay': Language use and antigay bias among heterosexual college students. Journal of Gay & Lesbian Social Services: The Quarterly Journal of Community & Clinical Practice 2012;24(3):241-259.

88. Chonody JM, Siebert DC, Rutledge SE. College students' attitudes toward gays and lesbians. Journal of Social Work Education 2009;45(3):499-512.

89. Chonody JM, Woodford MR, Brennan DJ, Newman B, Wang D. Attitudes Toward Gay Men and Lesbian Women Among Heterosexual Social Work Faculty. Journal of Social Work Education 2014;50(1):136-152.

90. Christensen JL, Miller LC, Appleby PR, Corsbie-Massay C, Godoy CG, Marsella SC, et al. Reducing shame in a game that predicts HIV risk reduction for young adult MSM: a randomized trial delivered nationally over the Web. J Int AIDS Soc 2013;16(3 Suppl 2):18716.

91. Chung YB, Szymanski DM. Racial and sexual identities of Asian American gay men. Journal of LGBT Issues in Counseling 2006;1(2):67-93.

92. Ciliberto J, Ferrari F. Interiorized homophobia, identity dynamics and gender typization. Hypothesizing a third gender role in Italian LGB individuals. J Homosex 2009;56(5):610-22.

93. Cirakoglu OC. Perception of homosexuality among Turkish university students: The roles of labels, gender, and prior contact. Journal of Social Psychology 2006;146(3):293-305.

94. Clow KA, Olson JM. Conceptual-Motor Compatibility and Homonegativity: Approaching and Avoiding Words Associated With Homosexuality. Canadian Journal of Behavioural Science 2010;42(4):222-233.

95. Cochran BN, Peavy KM, Cauce AM. Substance abuse treatment providers' explicit and implicit attitudes regarding sexual minorities. J Homosex 2007;53(3):181-207.

96. Cohen LA, Romberg E, Grace EG, Barnes DM. Attitudes of advanced dental education students toward individuals with AIDS. J Dent Educ 2005;69(8):896-900.

97. Collier KL, Bos HM, Sandfort TG. Intergroup contact, attitudes toward homosexuality, and the role of acceptance of gender non-conformity in young adolescents. J Adolesc 2012;35(4):899-907.

98. Conley CL. The development and initial validation of the COPLAG scale: Measuring the concerns of parents of lesbians and gays. Journal of Gay & Lesbian Social Services: The Quarterly Journal of Community & Clinical Practice 2011;23(1):30-52.

99. Connors J, Hely A. Attitudes toward people living with HIV/AIDS: A model of attitudes to illness. Journal of Applied Social Psychology 2007;37(1):124-130.

100. Coronges KA, Miller KA, Tamayo CI, Ender MG. A network evaluation of attitudes toward gays and lesbians among U. S. military cadets. Journal of Homosexuality 2013;60(11):1557-1580.

101. Costa PA, Davies M. Portuguese adolescents' attitudes toward sexual minorities: transphobia, homophobia, and gender role beliefs. J Homosex 2012;59(10):1424-42.

102. Costa PA, Pereira H, Leal I. Internalized homonegativity, disclosure, and acceptance of sexual orientation in a sample of Portuguese gay and bisexual men, and lesbian and bisexual women. Journal of Bisexuality 2013;13(2):229-244.

103. Cowan G, Heiple B, Marquez C, Khatchadourian D, McNevin M. Heterosexuals' attitudes toward hate crimes and hate speech against gays and lesbians: old-fashioned and modern heterosexism. J Homosex 2005;49(2):67-82.

104. Cox N, Dewaele A, van Houtte M, Vincke J. Stress-related growth, coming out, and internalized homonegativity in lesbian, gay, and bisexual youth. An examination of stress-related growth within the minority stress model. J Homosex 2011;58(1):117-37.

105. Cox N, Vanden Berghe W, Dewaele A, Vincke J. Acculturation strategies and mental health in gay, lesbian, and bisexual youth. J Youth Adolesc 2010;39(10):1199-210.

106. Cox S, Bimbi DS, Parsons JT. Examination of social contact on binegativity among lesbians and gay men. Journal of Bisexuality 2013;13(2):215-228.

107. Crisp C. Correlates of homophobia and use of gay affirmative practice among social workers. Journal of Human Behavior in the Social Environment 2006;14(4):119-143.

108. Cunningham GB, Melton EN. The moderating effects of contact with lesbian and gay friends on the relationships among religious fundamentalism, sexism, and sexual prejudice. Journal of Sex Research 2013;50(3-4):401-408.

109. Cunningham GB, Melton N. Prejudice against lesbian, gay, and bisexual coaches: The influence of race, religious fundamentalism, modern sexism, and contact with sexual minorities. Sociology of Sport Journal 2012;29(3):283-305.

110. Currie MR, Cunningham EG, Findlay BM. The Short Internalized Homonegativity Scale: examination of the factorial structure of a new measure of internalized homophobia. Educational & Psychological Measurement 2004;64(6):1053-1067.

111. D'Augelli AR, Grossman AH, Starks MT, Sinclair KO. Factors associated with parents' knowledge of gay, lesbian, and bisexual youths' sexual orientation. Journal of GLBT Family Studies 2010;6(2):178-198.

112. David S, Knight BG. Stress and coping among gay men: age and ethnic differences. Psychol Aging 2008;23(1):62-9.

113. Davies M. Correlates of negative attitudes toward gay men: sexism, male role norms, and male sexuality. J Sex Res 2004;41(3):259-66.

114. Davies M, Gilston J, Rogers P. Examining the relationship between male rape myth acceptance, female rape myth acceptance, victim blame, homophobia, gender roles, and ambivalent sexism. J Interpers Violence 2012;27(14):2807-23.

115. de la Rubia JM, de la O AV. Validación de la Escala Mexicana de Homofobia EHF-6. Psicología Iberoamericana 2011;19(1):80-88.

116. Delgado JB, Castro MC. Construction and validation of a subjective scale of stigma and discrimination (sisd) for the gay men and transgender women population in chile. Sexuality Research & Social Policy: A Journal of the NSRC 2014.

117. DeLonga K, Torres HL, Kamen C, Evans SN, Lee S, Koopman C, et al. Loneliness, Internalized Homophobia, and Compulsive Internet Use: Factors Associated with Sexual Risk Behavior among a Sample of Adolescent Males Seeking Services at a Community LGBT Center. Sexual Addiction & Compulsivity 2011;18(2):61-74.

118. Derlega VJ, Winstead BA, Brockington JE, Jr. AIDS stigma among inmates and staff in a USA state prison. Int J STD AIDS 2008;19(4):259-63.

119. Dermody N, Jones MK, Cumming SR. The failure of imagined contact in reducing explicit and implicit out-group prejudice toward male homosexuals. Current Psychology: A Journal for Diverse Perspectives on Diverse Psychological Issues 2013;32(3):261-274.

120. Dessel AB. Effects of intergroup dialogue: Public school teachers and sexual orientation prejudice. Small Group Research 2010;41(5):556-592.

121. Detenber BH, Cenite M, Ku MKY, Ong CPL, Tong HY, Yeow MLH. Singaporeans' attitudes toward lesbians and gay men and their tolerance of media portrayals of homosexuality. International Journal of Public Opinion Research 2007;19(3):367-379.

122. Detenber BH, Ho SS, Neo RL, Malik S, Cenite M. Influence of value predispositions, interpersonal contact, and mediated exposure on public attitudes toward homosexuals in Singapore. Asian Journal of Social Psychology 2013;16(3):181-196.

123. Dew BJ, Chaney MP. The relationship among sexual compulsivity, internalized homophobia, and HIV at-risk sexual behavior in gay and bisexual male users of Internet chat rooms. Sexual Addiction and Compulsivity 2005;12(4):259-273.

124. Diaz RM, Ayala G, Bein E. Sexual risk as an outcome of social oppression: data from a probability sample of Latino gay men in three U.S. cities. Cultur Divers Ethnic Minor Psychol 2004;10(3):255-67.

125. Dinkel S, Patzel B, McGuire MJ, Rolfs E, Purcell K. Measures of homophobia among nursing students and faculty: a Midwestern perspective. Int J Nurs Educ Scholarsh 2007;4:Article24.

126. Dowling KB, Rodger S, Cummings AL. Exploring attitudes of future educators about sexual minority youth. Alberta Journal of Educational Research 2007;53(4):401-413.

127. Dowshen N, Binns HJ, Garofalo R. Experiences of HIV-related stigma among young men who have sex with men. AIDS Patient Care STDS 2009;23(5):371-6.

128. Dragowski EA, Halkitis PN, Grossman AH, D'Augelli AR. Sexual orientation victimization and posttraumatic stress symptoms among lesbian, gay, and bisexual youth. Journal of Gay & Lesbian Social Services: The Quarterly Journal of Community & Clinical Practice 2011;23(2):226-249.

129. Dudley MG, Rostosky SS, Korfhage BA, Zimmerman RS. Correlates of high-risk sexual behavior among young men who have sex with men. AIDS Educ Prev 2004;16(4):328-40.

130. Dunjic-Kostic B, Pantovic M, Vukovic V, Randjelovic D, Totic-Poznanovic S, Damjanovic A, et al. Knowledge: a possible tool in shaping medical professionals' attitudes towards homosexuality. Psychiatr Danub 2012;24(2):143-51.

131. Durso LE, Meyer IH. Patterns and Predictors of Disclosure of Sexual Orientation to Healthcare Providers among Lesbians, Gay Men, and Bisexuals. Sex Res Social Policy 2013;10(1):35-42.

132. Edwards KM, Sylaska KM. The perpetration of intimate partner violence among LGBTQ college youth: the role of minority stress. J Youth Adolesc 2013;42(11):1721-31.

133. Einbinder SD, Fiechter S, Sheridan DA, Miller DL. Social work educators’ attitudes toward gay men and lesbians: A national assessment. Journal of Gay & Lesbian Social Services: The Quarterly Journal of Community & Clinical Practice 2012;24(2):173-200.

134. Eldridge J, Johnson P. The relationship between old-fashioned and modern heterosexism to social dominance orientation and structural violence. J Homosex 2011;58(3):382-401.

135. Eliason MJ, Hughes T. Treatment counselor's attitudes about lesbian, gay, bisexual, and transgendered clients: urban vs. rural settings. Subst Use Misuse 2004;39(4):625-44.

136. Ensign KA, Yiamouyiannis A, White KM, Ridpath BD. Athletic trainers' attitudes toward lesbian, gay, and bisexual National Collegiate Athletic Association student-athletes. Journal of Athletic Training 2011;46(1):69-75.

137. Erich S, Tittsworth J, Meier SLC, Lerman T. Transsexuals of Color: Perceptions of discrimination based on transsexual status and race/ethnicity status. Journal of GLBT Family Studies 2010;6(3):294-314.

138. Estrada F, Rigali-Oiler M, Arciniega GM, Tracey TJ. Machismo and Mexican American men: an empirical understanding using a gay sample. J Couns Psychol 2011;58(3):358-67.

139. Falomir-Pichastor JM, Martinez C, Paterna C. Gender-role's attitude, perceived similarity, and sexual prejudice against gay men. Span J Psychol 2010;13(2):841-8.

140. Falomir-Pichastor JM, Mugny G. “I'm not gay…I'm a real man!”: Heterosexual men's gender self-esteem and sexual prejudice. Personality and Social Psychology Bulletin 2009;35(9):1233-1243.

141. Feinstein BA, Goldfried MR, Davila J. The relationship between experiences of discrimination and mental health among lesbians and gay men: An examination of internalized homonegativity and rejection sensitivity as potential mechanisms. J Consult Clin Psychol 2012;80(5):917-27.

142. Fernandez MI, Jacobs RJ, Warren JC, Sanchez J, Bowen GS. Drug use and Hispanic men who have sex with men in South Florida: implications for intervention development. AIDS Educ Prev 2009;21(5 Suppl):45-60.

143. Fingerhut AW. Straight allies: What predicts heterosexuals' alliance with the LGBT community? Journal of Applied Social Psychology 2011;41(9):2230-2248.

144. Finneran C, Chard A, Sineath C, Sullivan P, Stephenson R. Intimate Partner Violence and Social Pressure among Gay Men in Six Countries. West J Emerg Med 2012;13(3):260-71.

145. Finneran C, Stephenson R. Gay and Bisexual Men's Perceptions of Police Helpfulness in Response to Male-Male Intimate Partner Violence. West J Emerg Med 2013;14(4):354-62.

146. Finneran C, Stephenson R. Intimate partner violence, minority stress, and sexual risk-taking among U.S. men who have sex with men. J Homosex 2014;61(2):288-306.

147. Fisher LE, Banik S. College major, gender and heterosexism reconsidered under more controlled conditions. J LGBT Health Res 2007;3(1):49-53.

148. Fisher Raymond H, Chen YH, Stall RD, McFarland W. Adolescent Experiences of Discrimination, Harassment, Connectedness to Community and Comfort with Sexual Orientation Reported by Adult Men Who have Sex with Men as a Predictor of Adult HIV Status. AIDS Behav 2009:1-7.

149. Flebus GB, Montano A. The Multifactor Internalized Homophobia Inventory. TPM-Testing, Psychometrics, Methodology in Applied Psychology 2012;19(3):219-240.

150. Flores SA, Mansergh G, Marks G, Guzman R, Colfax G. Gay identity-related factors and sexual risk among men who have sex with men in San Francisco. AIDS Educ Prev 2009;21(2):91-103.

151. Folch C, Munoz R, Zaragoza K, Casabona J. Sexual risk behaviour and its determinants among men who have sex with men in Catalonia, Spain. Euro Surveill 2009;14(47).

152. Fredriksen-Goldsen KI, Cook-Daniels L, Kim HJ, Erosheva EA, Emlet CA, Hoy-Ellis CP, et al. Physical and Mental Health of Transgender Older Adults: An At-Risk and Underserved Population. Gerontologist 2013.

153. Fredriksen-Goldsen KI, Emlet CA, Kim HJ, Muraco A, Erosheva EA, Goldsen J, et al. The physical and mental health of lesbian, gay male, and bisexual (LGB) older adults: the role of key health indicators and risk and protective factors. Gerontologist 2013;53(4):664-75.

154. Freis SD, Gurung RAR. A Facebook analysis of helping behavior in online bullying. Psychology of Popular Media Culture 2013;2(1):11-19.

155. Frias-Navarro D, Monterde IBH. A scale on beliefs about children's adjustment in same-sex families: reliability and validity. J Homosex 2012;59(9):1273-88.

156. Frost DM, Meyer IH. Internalized Homophobia and Relationship Quality among Lesbians, Gay Men, and Bisexuals. J Couns Psychol 2009;56(1):97-109.

157. Frost DM, Parsons JT, Nanin JE. Stigma, concealment and symptoms of depression as explanations for sexually transmitted infections among gay men. J Health Psychol 2007;12(4):636-40.

158. Furnham A, Saito K. A cross-cultural study of attitudes toward and beliefs about, male homosexuality. J Homosex 2009;56(3):299-318.

159. Gaines SO, Henderson MC, Kim M, Gilstrap S, Yi J, Rusbult CE, et al. Cultural value orientations, internalized homophobia, and accommodation in romantic relationships. J Homosex 2005;50(1):97-117.

160. Gandy ME, McCarter SA, Portwood SG. Service providers' attitudes toward LGBTQ youth. Residential Treatment for Children & Youth 2013;30(3):168-186.

161. Gates TG, Mitchell CG. Workplace stigma-related experiences among lesbian, gay, and bisexual workers: Implications for social policy and practice. Journal of Workplace Behavioral Health 2013;28(3):159-171.

162. Gato J, Fontaine AM, Cameiro NS. Escala multidimensional de atitudes face a lésbicas e a gays: Construçäo e validação preliminar. Paidéia 2012;22(51):11-20.

163. Gattis MN. An Ecological Systems Comparison Between Homeless Sexual Minority Youths and Homeless Heterosexual Youths. J Soc Serv Res 2013;39(1):38-49.

164. Gelbal S, Duyan V. Attitudes of university students toward lesbians and gay men in Turkey. Sex Roles 2006;55(7-8):573-579.

165. Gencoz T, Yuksel M. Psychometric properties of the Turkish version of the internalized homophobia scale. Arch Sex Behav 2006;35(5):597-602.

166. Gerhardstein KR, Anderson VN. There’s more than meets the eye: Facial appearance and evaluations of transsexual people. Sex Roles 2010;62(5-6):361-373.

167. Gilbert PA, Rhodes SD. HIV testing among immigrant sexual and gender minority Latinos in a US region with little historical Latino presence. AIDS Patient Care STDS 2013;27(11):628-36.

168. Gold SD, Feinstein BA, Skidmore WC, Marx BP. Childhood physical abuse, internalized homophobia, and experiential avoidance among lesbians and gay men. Psychological Trauma: Theory, Research, Practice, and Policy 2011;3(1):50-60.

169. Gold SD, Marx BP, Lexington JM. Gay male sexual assault survivors: the relations among internalized homophobia, experiential avoidance, and psychological symptom severity. Behav Res Ther 2007;45(3):549-62.

170. Goldberg AE, Smith JZ. Stigma, social context, and mental health: lesbian and gay couples across the transition to adoptive parenthood. J Couns Psychol 2011;58(1):139-50.

171. Goldberg AE, Smith JZ. Work conditions and mental health in lesbian and gay dual‐earner parents. Family Relations: An Interdisciplinary Journal of Applied Family Studies 2013;62(5):727-740.

172. Golub SA, Rendina HJ, Gamarel KE. Identity-related growth and loss in a sample of HIV-positive gay and bisexual men: initial scale development and psychometric evaluation. AIDS Behav 2013;17(2):748-59.

173. Gomleksiz M, Poyrazli S, Vural RA. Discriminatory attitudes: A scale development in Turkish. Psychological Reports 2008;103(3):921-930.

174. Goodman MB, Moradi B. Attitudes and behaviors toward lesbian and gay persons: Critical correlates and mediated relations. Journal of Counseling Psychology 2008;55(3):371-384.

175. Goodnight BL C, SL, Parrott DJ, & Peterson JL. Effects of Masculinity, Authoritarianism, And Prejudice on Antigay Aggression: A Path Analysis of Gender-Role Enforcement. Psychology of Men & Masculinity 2014;15(4):437-444.

176. Gordon AR, Meyer IH. Gender nonconformity as a target of prejudice, discrimination, and violence against LGB individuals. J LGBT Health Res 2007;3(3):55-71.

177. Gormley B, Lopez FG. Authoritarian and homophobic attitudes: gender and adult attachment style differences. J Homosex 2010;57(4):525-38.

178. Green MS, Murphy MJ, Blumer M, Palmanteer D. Marriage and family therapists’ comfort level working with gay and lesbian individuals, couples, and families. American Journal of Family Therapy 2009;37(2):159-168.

179. Greene K, Banerjee SC. Disease-related stigma: comparing predictors of AIDS and cancer stigma. J Homosex 2006;50(4):185-209.

180. Greentree S, Lewis V. Male body image and its relationship to sexual preference and homophobia. Pakistan Journal of Psychological Research 2011;26(2):105-126.

181. Gromer JM, Campbell MH, Gomory T, Maynard DM. Sexual prejudice among Barbadian university students. Journal of Gay & Lesbian Social Services: The Quarterly Journal of Community & Clinical Practice 2013;25(4):399-419.

182. Grosskopf NA, Levasseur MT, Glaser DB. Use of the Internet and Mobile-Based "Apps" for Sex-Seeking Among Men Who Have Sex With Men in New York City. Am J Mens Health 2014.

183. Guo Y, Li X, Liu Y, Jiang S, Tu X. Disclosure of same-sex behavior by young Chinese migrant men: context and correlates. Psychol Health Med 2014;19(2):190-200.

184. Gursimsek I. Sexual education and teacher candidates' attitudes toward sexuality. Australian Journal of Guidance & Counselling 2010;20(1):81-90.

185. Guth LJ, Lopez DF, Rojas J, Clements KD, Tyler JM. Experiential versus rational training: a comparison of student attitudes toward homosexuality. J Homosex 2004;48(2):83-102.

186. Guzman MG, Ortiz Mdel C, Torres RR, Alfonso JT. Attitudes towards homosexual and lesbians among Puerto Rican Public Health graduate students. P R Health Sci J 2007;26(3):221-4.

187. Halkitis PN, Kapadia F, Siconolfi DE, Moeller RW, Figueroa RP, Barton SC, et al. Individual, psychosocial, and social correlates of unprotected anal intercourse in a new generation of young men who have sex with men in New York City. Am J Public Health 2013;103(5):889-95.

188. Hamilton CJ, Mahalik JR. Minority stress, masculinity, and social norms predicting gay men's health risk behaviors. Journal of Counseling Psychology 2009;56(1):132-141.

189. Harbin JJ, Leach MM, Eells GT. Homonegativism and sexual orientation matching in counseling supervision. Counselling Psychology Quarterly 2008;21(1):61-73.

190. Harris JI, Cook SW, Kashubeck-West S. Religious attitudes, internalized homophobia, and identity in gay and lesbian adults. Journal of Gay & Lesbian Mental Health 2008;12(3):205-225.

191. Hatzenbuehler ML, Dovidio JF, Nolen-Hoeksema S, Phills CE. An Implicit Measure of Anti-Gay Attitudes: Prospective Associations with Emotion Regulation Strategies and Psychological Distress. J Exp Soc Psychol 2009;45(6):1316-1320.

192. Hatzenbuehler ML, McLaughlin KA. Structural stigma and hypothalamic-pituitary-adrenocortical axis reactivity in lesbian, gay, and bisexual young adults. Ann Behav Med 2014;47(1):39-47.

193. Hatzenbuehler ML, Nolen-Hoeksema S, Dovidio J. How does stigma "get under the skin"?: the mediating role of emotion regulation. Psychol Sci 2009;20(10):1282-9.

194. Heath J, Goggin K. Attitudes towards male homosexuality, bisexuality, and the down low lifestyle: Demographic differences and HIV implications. Journal of Bisexuality 2009;9(1):17-31.

195. Hegarty P, Pratto F, Lemieux AF. Heterosexist ambivalence and heterocentric norms: Drinking in intergroup discomfort. Group Processes & Intergroup Relations 2004;7(2):119-130.

196. Heinke T, Carlson TS, McGeorge CR. Homophobia and clinical competency: an exploration of couple and family therapists' beliefs. Journal of Couple & Relationship Therapy 2009;8(4):325-342.

197. Hequembourg AL, Bimbi D, Parsons JT. Sexual victimization and health-related indicators among sexual minority men. J LGBT Issues Couns 2011;5(1):2-20.

198. Hequembourg AL, Dearing RL. Exploring shame, guilt, and risky substance use among sexual minority men and women. J Homosex 2013;60(4):615-38.

199. Hequembourg AL, Parks KA, Collins RL, Hughes TL. Sexual Assault Risks Among Gay and Bisexual Men. J Sex Res 2014.

200. Herbstrith JC, Tobin RM, Hesson-McInnis MS, Joel Schneider W. Preservice teacher attitudes toward gay and lesbian parents. Sch Psychol Q 2013;28(3):183-94.

201. Herek GM. Hate crimes and stigma-related experiences among sexual minority adults in the United States: prevalence estimates from a national probability sample. J Interpers Violence 2009;24(1):54-74.

202. Herek GM, Gillis JR, Cogan JC. Internalized stigma among sexual minority adults: Insights from a social psychological perspective. Journal of Counseling Psychology 2009;56(1):32-43.

203. Herek GM, Gonzalez-Rivera M. Attitudes toward homosexuality among U.S. residents of Mexican descent. J Sex Res 2006;43(2):122-35.

204. Hetzel CJ. Exploring the relationship between public opinion and personal attitudes and behavior toward lesbians and gay men: social conformity revisited. J Homosex 2011;58(10):1421-41.

205. Higgins DJ. Differences between previously married and never married 'gay' men: family background, childhood experiences and current attitudes. J Homosex 2004;48(1):19-41.

206. Hightow-Weidman LB, Phillips G, 2nd, Jones KC, Outlaw AY, Fields SD, Smith JC. Racial and sexual identity-related maltreatment among minority YMSM: prevalence, perceptions, and the association with emotional distress. AIDS Patient Care STDS 2011;25 Suppl 1:S39-45.

207. Hilliard RE. A social and historical perspective of the San Francisco Gay Men's Chorus. J Homosex 2008;54(4):345-61.

208. Holland L, Matthews TL, Schott MR. "That's so gay!" Exploring college students' attitudes toward the LGBT population. J Homosex 2013;60(4):575-95.

209. Holloway IW, Traube DE, Rice E, Schrager SM, Palinkas LA, Richardson J, et al. Community and Individual Factors Associated with Cigarette Smoking Among Young Men Who Have Sex With Men. J Res Adolesc 2012;22(2):199-205.

210. Hong Y, Fang X, Li X, Liu Y, Li M, Tai-Seale T. Self-perceived stigma, depressive symptoms, and suicidal behaviors among female sex workers in China. J Transcult Nurs 2010;21(1):29-34.

211. Hooghe M. The impact of gendered friendship patterns on the prevalence of homophobia among Belgian late adolescents. Archives of Sexual Behavior 2011;40(3):543-550.

212. Hooghe M, Claes E, Harell A, Quintelier E, Dejaeghere Y. Anti-gay sentiment among adolescents in Belgium and Canada: a comparative investigation into the role of gender and religion. J Homosex 2010;57(3):384-400.

213. Hooghe M, Meeusen C. Homophobia and the transition to adulthood: a three year panel study among Belgian late adolescents and young adults, 2008-2011. J Youth Adolesc 2012;41(9):1197-207.

214. Horn SS, Heinze J. 'She can't help it, she was born that way': Adolescents' beliefs about the origins of homosexuality and sexual prejudice. Anales de Psicología 2011;27(3):688-697.

215. Horn SS, Szalach LA. School differences in heterosexual students' attitudes about homosexuality and prejudice based on sexual orientation. European Journal of Developmental Science 2009;3(1):64-79.

216. Horn SS, Szalacha LA, Drill K. Schooling, sexuality, and rights: An investigation of heterosexual students' social cognition regarding sexual orientation and the rights of gay and lesbian peers in school. Journal of Social Issues 2008;64(4):791-813.

217. Horne SG, Biss WJ. Sexual Satisfaction as More Than a Gendered Concept: The Roles of Psychological Well-Being and Sexual Orientation. Journal of Constructivist Psychology 2005;18(1):25-38.

218. Horner B, McManus A, Comfort J, Freijah R, Lovelock G, Hunter M, et al. How prepared is the retirement and residential aged care sector in Western Australia for older non-heterosexual people? Qual Prim Care 2012;20(4):263-74.

219. Hosseinzadeh H, Hossain SZ, Niknami S. Levels and functions of HIV/AIDS stigma within the Iranian community living in the Sydney metropolitan area. Health Education Journal 2012;71(1):115-128.

220. House AS, Van Horn E, Coppeans C, Stepleman LM. Interpersonal trauma and discriminatory events as predictors of suicidal and nonsuicidal self-injury in gay, lesbian, bisexual, and transgender persons. Traumatology 2011;17(2):75-85.

221. Hu X, Wang Y. LGB identity among young Chinese: The influence of traditional culture. Journal of Homosexuality 2013;60(5):667-684.

222. Hudepohl AD, Parrott DJ, Zeichner A. Heterosexual men's anger in response to male homosexuality: effects of erotic and non-erotic depictions of male-male intimacy and sexual prejudice. J Homosex 2010;57(8):1022-38.

223. Huebner DM, Davis MC. Perceived antigay discrimination and physical health outcomes. Health Psychol 2007;26(5):627-34.

224. Huebner DM, Kegeles SM, Rebchook GM, Peterson JL, Neilands TB, Johnson WD, et al. Social Oppression, Psychological Vulnerability, and Unprotected Intercourse Among Young Black Men Who Have Sex With Men. Health Psychol 2013.

225. Huebner DM, Nemeroff CJ, Davis MC. Do hostility and neuroticism confound associations between perceived discrimination and depressive symptoms? Journal of Social and Clinical Psychology 2005;24(5):723-740.

226. Hunt CJ, Gonsalkorale K, Nosek BA. Links between psychosocial variables and body dissatisfaction in homosexual men: Differential relations with the drive for muscularity and the drive for thinness. International Journal of Men's Health 2012;11(2):127-136.

227. Hussey HD, Bisconti TL. Interventions to reduce sexual minority stigma in sororities. J Homosex 2010;57(4):566-87.

228. Hylton ME. Online versus classroom-based instruction: A comparative study of learning outcomes in a diversity course. The Journal of Baccalaureate Social Work 2006;11(2):102-114.

229. Irwin JA, Coleman JD, Fisher CM, Marasco VM. Correlates of Suicide Ideation Among LGBT Nebraskans. J Homosex 2013.

230. Israel T, Hackett G. Counselor Education on Lesbian, Gay, and Bisexual Issues: Comparing Information and Attitude Exploration. Counselor Education and Supervision 2004;43(3):179-191.

231. Jacobs RJ, Fernandez MI, Ownby RL, Bowen GS, Hardigan PC, Kane MN. Factors associated with risk for unprotected receptive and insertive anal intercourse in men aged 40 and older who have sex with men. AIDS Care 2010;22(10):1204-11.

232. Jacobs RJ, Kane MN. Correlates of loneliness in midlife and older gay and bisexual men. Journal of Gay & Lesbian Social Services: The Quarterly Journal of Community & Clinical Practice 2012;24(1):40-61.

233. James CA, Schwartz DR, Roberts KE, Hart TA, Loutfy MR, Myers T, et al. Childhood emotional abuse and psychological distress in gay and bisexual men. Journal of Aggression, Maltreatment and Trauma 2012;21(8):851-869.

234. James W, Griffiths B, Pedersen A. The “making and unmaking” of prejudice against Australian Muslims and gay men and lesbians: The role of religious development and fundamentalism. International Journal for the Psychology of Religion 2011;21(3):212-227.

235. Jayaratne TE, Ybarra O, Sheldon JP, Brown TN, Feldbaum M, Pfeffer C, et al. White Americans' Genetic Lay Theories of Race Differences and Sexual Orientation: Their Relationship with Prejudice toward Blacks, and Gay Men and Lesbians. Group Process Intergroup Relat 2006;9(1).

236. Jefferson SD, Bramlett F. The moderating roles of gender and anti-gay prejudice in explaining stigma by association in male dyads. J Homosex 2010;57(3):401-14.

237. Jellison WA, McConnell AR, Gabriel S. Implicit and Explicit Measures of Sexual Orientation Attitudes: Ingroup Preferences and Related Behaviors and Beliefs Among Gay and Straight Men. Personality and Social Psychology Bulletin 2004;30(5):629-642.

238. Jenkins M, Lambert EG, Baker DN. The attitudes of Black and White college students toward gays and lesbians. Journal of Black Studies 2009;39(4):589-613.

239. Jin H, Earnshaw VA, Wickersham JA, Kamarulzaman A, Desai MM, John J, et al. An assessment of health-care students' attitudes toward patients with or at high risk for HIV: implications for education and cultural competency. AIDS Care 2014.

240. Johnson MO, Carrico AW, Chesney MA, Morin SF. Internalized heterosexism among HIV-positive, gay-identified men: implications for HIV prevention and care. J Consult Clin Psychol 2008;76(5):829-39.

241. Kamen C, Burns M, Beach SR. Minority stress in same-sex male relationships: when does it impact relationship satisfaction? J Homosex 2011;58(10):1372-90.

242. Kamise Y. Occupational stigma and coping strategies of women engaged in the commercial sex industry: A study on the perception of 'kyaba-cula hostesses' in Japan. Sex Roles 2013;69(1-2):42-57.

243. Kan RWM, Au KP, Chan WK, Cheung LWM, Lam CYY, Liu HHW, et al. Homophobia in medical students of the University of Hong Kong. Sex Education 2009;9(1):65-80.

244. Kappler S, Hancock KA, Plante TG. Roman catholic gay priests: Internalized homophobia, sexual identity, and psychological well-being. Pastoral Psychology 2012.

245. Kashubeck-West S, Szymanski DM. Risky sexual behavior in gay and bisexual men: Internalized heterosexism, sensation seeking, and substance use. The Counseling Psychologist 2008;36(4):595-614.

246. Keiller SW. Abstract reasoning as a predictor of attitudes toward gay men. J Homosex 2010;57(7):914-27.

247. Keiller SW. Masculine norms as correlates of heterosexual men’s attitudes toward gay men and lesbian women. Psychology of Men & Masculinity 2010;11(1):38-52.

248. Kelleher C. Minority stress and health: implications for lesbian, gay, bisexual, transgender, and questioning (LGBTQ) young people. Counselling Psychology Quarterly 2009;22(4):373-379.

249. Kelley TM, Robertson RA. Relational aggression and victimization in gay male relationships: the role of internalized homophobia. Aggress Behav 2008;34(5):475-85.

250. Kelly BC, Bimbi DS, Izienicki H, Parsons JT. Stress and coping among HIV-positive barebackers. AIDS Behav 2009;13(4):792-7.

251. Kimmel SB, Mahalik JR. Body image concerns of gay men: the roles of minority stress and conformity to masculine norms. J Consult Clin Psychol 2005;73(6):1185-90.

252. King EJ, Maman S, Bowling JM, Moracco KE, Dudina V. The influence of stigma and discrimination on female sex workers' access to HIV services in St. Petersburg, Russia. AIDS Behav 2013;17(8):2597-603.

253. King ME, Winter S, Webster B. Contact reduces transprejudice: a study on attitudes towards transgenderism and transgender civil rights in Hong Kong. International Journal of Sexual Health 2009;21(1):17-34.

254. King SD, Orel N. Midlife and older gay men living with HIV/AIDS: The influence of resiliency and psychosocial stress factors on health needs. Journal of Gay & Lesbian Social Services: The Quarterly Journal of Community & Clinical Practice 2012;24(4):346-370.

255. Kissinger DB, Lee SM, Twitty L, Kisner H. Impact of family environment on future mental health professionals' attitudes toward lesbians and gay men. J Homosex 2009;56(7):894-920.

256. Klein O, Livingston RW, Snyder M. Être ou ne pas être politiquement correct? la relation entre préjugé et expression d'impressions stéréotypées en fonction du self-monitoring. Cahiers Internationaux de Psychologie Sociale 2005;67-68:55-64.

257. Kooyman LE. Predictors of high-risk sexual behavior among gay men and men who have sex with men. Journal of LGBT Issues in Counseling 2008;2(4):285-307.

258. Korfhage BA, Rostosky SS. Psychology graduate students' attitudes toward lesbians and gay men. J Homosex 2006;51(4):145-159.

259. Korhonen T, Kylma J, Houtsonen J, Valimaki M, Suominen T. University students' knowledge of, and attitudes towards, HIV and AIDS, homosexuality and sexual risk behaviour: a questionnaire survey in two Finnish universities. J Biosoc Sci 2012;44(6):661-75.

260. Kralovec K, Fartacek C, Fartacek R, Ploderl M. Religion and suicide risk in lesbian, gay and bisexual austrians. J Relig Health 2014;53(2):413-23.

261. Kuhns LM, Vazquez R, Ramirez-Valles J. Researching special populations: retention of Latino gay and bisexual men and transgender persons in longitudinal health research. Health Educ Res 2008;23(5):814-25.

262. Kulik L. Transmission of Attitudes Regarding Family Life From Parents to Adolescents in Israel. Families in Society 2004;85(3):345-353.

263. Kuyper L, Fokkema T. Loneliness among older lesbian, gay, and bisexual adults: the role of minority stress. Arch Sex Behav 2010;39(5):1171-80.

264. Kuyper L, Vanwesenbeeck I. Examining sexual health differences between lesbian, gay, bisexual, and heterosexual adults: the role of sociodemographics, sexual behavior characteristics, and minority stress. J Sex Res 2011;48(2-3):263-74.

265. Kwok DK, Wu J, Shardlow SM. Attitudes toward lesbians and gay men among Hong Kong Chinese social work students. Journal of Social Work Education 2013;49(2):337-352.

266. Kwon P, Hugelshofer DS. Lesbian, gay, and bisexual speaker panels lead to attitude change among heterosexual college students. Journal of Gay & Lesbian Social Services: The Quarterly Journal of Community & Clinical Practice 2012;24(1):62-79.

267. Lai CK, Haidt J, Nosek BA. Moral elevation reduces prejudice against gay men. Cogn Emot 2013.

268. Lambert EG, Ventura LA, Hall DE, Cluse-Tolar T. College students' views on gay and lesbian issues: does education make a difference. J Homosex 2006;50(4):1-30.

269. L'Archevêque A, Julien D. Intégration des identités homosexuelle et paternelle chez les pères gais. Canadian Journal of Behavioural Science/Revue canadienne des sciences du comportement 2011.

270. Latner JD, O'Brien KS, Durso LE, Brinkman LA, MacDonald T. Weighing obesity stigma: the relative strength of different forms of bias. Int J Obes (Lond) 2008;32(7):1145-52.

271. Lau JT, Choi KC, Tsui HY, Su X. Associations between stigmatization toward HIV-related vulnerable groups and similar attitudes toward people living with HIV/AIDS: branches of the same tree? AIDS Care 2007;19(10):1230-40.

272. Lea T, de Wit J, Reynolds R. Minority Stress in Lesbian, Gay, and Bisexual Young Adults in Australia: Associations with Psychological Distress, Suicidality, and Substance Use. Arch Sex Behav 2014.

273. Lemm KM. Positive associations among interpersonal contact, motivation, and implicit and explicit attitudes toward gay men. J Homosex 2006;51(2):79-99.

274. Levin L, Peled E. The Attitudes Toward Prostitutes and Prostitution Scale: A New Tool for Measuring Public Attitudes Toward Prostitutes and Prostitution. Research on Social Work Practice 2011;21(5):582-593.

275. Lewis AJ, White J. Brief report: The defense mechanisms of homophobic adolescent males: A descriptive discriminant analysis. J Adolesc 2009;32(2):435-41.

276. Li MJ, Distefano A, Mouttapa M, Gill JK. Bias-motivated bullying and psychosocial problems: implications for HIV risk behaviors among young men who have sex with men. AIDS Care 2014;26(2):246-56.

277. Liang CTH, Alimo C. The Impact of White Heterosexual Students' Interactions on Attitudes Toward Lesbian, Gay and Bisexual People: A Longitudinal Study. Journal of College Student Development 2005;46(3):237-250.

278. Liddle BJ, Luzzo DA, Hauenstein AL, Schuck K. Construction and Validation of the Lesbian, Gay, Bisexual, and Transgendered Climate Inventory. Journal of Career Assessment 2004;12(1):33-50.

279. Lin YJ, Israel T. A computer-based intervention to reduce internalized heterosexism in men. J Couns Psychol 2012;59(3):458-64.

280. Lingiardi V, Baiocco R, Nardelli N. Measure of internalized sexual stigma for lesbians and gay men: a new scale. J Homosex 2012;59(8):1191-210.

281. Lingiardi V, Falanga S, D'Augelli AR. The evaluation of homophobia in an Italian sample. Arch Sex Behav 2005;34(1):81-93.

282. Liu H, Feng T, Ha T, Liu H, Cai Y, Liu X, et al. Chinese Culture, Homosexuality Stigma, Social Support and Condom Use: A Path Analytic Model. Stigma Res Action 2011;1(1):27-35.

283. Liu H, Feng T, Rhodes AG, Liu H. Assessment of the Chinese version of HIV and homosexuality related stigma scales. Sex Transm Infect 2009;85(1):65-9.

284. Liu SH, Srikrishnan AK, Zelaya CE, Solomon S, Celentano DD, Sherman SG. Measuring perceived stigma in female sex workers in Chennai, India. AIDS Care 2011;23(5):619-27.

285. Logie CH, Newman PA, Chakrapani V, Shunmugam M. Adapting the minority stress model: associations between gender non-conformity stigma, HIV-related stigma and depression among men who have sex with men in South India. Soc Sci Med 2012;74(8):1261-8.

286. Lombardi E. Varieties of transgender/transsexual lives and their relationship with transphobia. J Homosex 2009;56(8):977-92.

287. Long SL, Mollen D, Smith NG. College women’s attitudes toward sex workers. Sex Roles 2012;66(1-2):117-127.

288. Long W, Millsap CA. Fear of AIDS and Homophobia Scales in an ethnic population of university students. J Soc Psychol 2008;148(5):637-40.

289. Lottes IL, Grollman EA. Conceptualization and assessment of homonegativity. International Journal of Sexual Health 2010;22(4):219-233.

290. Luu TD, Bartsch RA. Relationship between acculturation and attitudes toward gay men and lesbians in the Vietnamese American community. Journal of Applied Social Psychology 2011;41(11):2621-2633.

291. Lyons A, Pitts M, Grierson J. Factors related to positive mental health in a stigmatized minority: an investigation of older gay men. J Aging Health 2013;25(7):1159-81.

292. Lyons CJ. Stigma or Sympathy? Attributions of Fault to Hate Crime Victims and Offenders. Social Psychology Quarterly 2006;69(1):39-59.

293. Lyons PM, Jr., DeValve MJ, Garner RL. Texas police chiefs' attitudes toward gay and lesbian police officers. Police Quarterly 2008;11(1):102-117.

294. Magnus M, Herwehe J, Murtaza-Rossini M, Reine P, Cuffie D, Gruber D, et al. Linking and retaining HIV patients in care: the importance of provider attitudes and behaviors. AIDS Patient Care STDS 2013;27(5):297-303.

295. Mahaffey AL, Bryan A, Hutchison KE. Using Startle Eye Blink to Measure the Affective Component of Antigay Bias. Basic and Applied Social Psychology 2005;27(1):37-45.

296. Malcomnson KM, Christopher AN, Franzen T, Keyes BJ. The Protestant work ethic, religious beliefs, and homonegative attitudes. Mental Health, Religion and Culture 2006;9(5):435-447.

297. Malley M, Tasker F. Significant and other: Systemic family therapists on lesbians and gay men. Journal of Family Therapy 2004;26(2):193-212.

298. Mange J, Lepastourel N. Gender effect and prejudice: when a salient female norm moderates male negative attitudes toward homosexuals. J Homosex 2013;60(7):1035-53.

299. Marsh T, Brown J. Homonegativity and its relationship to religiosity, nationalism and attachment style. J Relig Health 2011;50(3):575-91.

300. Martinez P, Barsky A, Singleton S. Exploring queer consciousness among social workers. Journal of Gay & Lesbian Social Services: The Quarterly Journal of Community & Clinical Practice 2011;23(2):296-315.

301. Masser B, Moffat KB. With friends like these. . .the role of prejudice and situational norms on discriminatory helping behavior. J Homosex 2006;51(2):121-38.

302. Massey SG. Polymorphous prejudice: liberating the measurement of heterosexuals' attitudes toward lesbians and gay men. J Homosex 2009;56(2):147-72.

303. Mata J, Ghavami N, Wittig MA. Understanding Gender Differences in Early Adolescents' Sexual Prejudice. J Early Adolesc 2010;30(1):50-75.

304. Matharu K, Kravitz RL, McMahon GT, Wilson MD, Fitzgerald FT. Medical students' attitudes toward gay men. BMC Med Educ 2012;12:71.

305. Mavor KI, Gallois C. Social group and moral orientation factors as mediators of religiosity and multiple attitude targets. Journal for the Scientific Study of Religion 2008;47(3):361-377.

306. Mayer KH, Wang L, Koblin B, Mannheimer S, Magnus M, del Rio C, et al. Concomitant socioeconomic, behavioral, and biological factors associated with the disproportionate HIV infection burden among Black men who have sex with men in 6 U.S. cities. PLoS One 2014;9(1):e87298.

307. McAdams-Mahmoud A, Stephenson R, Rentsch C, Cooper H, Arriola KJ, Jobson G, et al. Minority Stress in the Lives of Men Who Have Sex With Men in Cape Town, South Africa. J Homosex 2014.

308. McCabe SE, Bostwick WB, Hughes TL, West BT, Boyd CJ. The relationship between discrimination and substance use disorders among lesbian, gay, and bisexual adults in the United States. Am J Public Health 2010;100(10):1946-52.

309. Meaney GJ, Rye BJ. Gendered egos: attitude functions and gender as predictors of homonegativity. J Homosex 2010;57(10):1274-302.

310. Mellinger C, Levant RF. Moderators of the relationship between masculinity and sexual prejudice in men: friendship, gender self-esteem, same-sex attraction, and religious fundamentalism. Arch Sex Behav 2014;43(3):519-30.

311. Miller AK, Wagner MM, Hunt AN. Parsimony in personality: predicting sexual prejudice. J Homosex 2012;59(2):201-14.

312. Mireshghi SI, Matsumoto D. Perceived cultural attitudes toward homosexuality and their effects on Iranian and American sexual minorities. Cultur Divers Ethnic Minor Psychol 2008;14(4):372-6.

313. Mohipp C, Morry MM. The Relationship of Symbolic Beliefs and Prior Contact to Heterosexuals' Attitudes Toward Gay Men and Lesbian Women. Canadian Journal of Behavioural Science/Revue canadienne des sciences du comportement 2004;36(1):36-44.

314. Mohr JJ, Daly CA. Sexual minority stress and changes in relationship quality in same-sex couples. Journal of Social & Personal Relationships 2008;25(6):989-1007.

315. Mohr JJ, Fassinger RE. Sexual orientation identity and romantic relationship quality in same-sex couples. Pers Soc Psychol Bull 2006;32(8):1085-99.

316. Mohr JJ, Kendra MS. Revision and extension of a multidimensional measure of sexual minority identity: the Lesbian, Gay, and Bisexual Identity Scale. J Couns Psychol 2011;58(2):234-45.

317. Molina Y, Ramirez-Valles J. HIV/AIDS stigma: measurement and relationships to psycho-behavioral factors in Latino gay/bisexual men and transgender women. AIDS Care 2013;25(12):1559-68.

318. Monto MA, Supinski J. Discomfort with Homosexuality: A New Measure Captures Differences in Attitudes Toward Gay Men and Lesbians. J Homosex 2013.

319. Moradi B, Van Den Berg JJ, Epting FR. Intrapersonal and interpersonal manifestations of antilesbian and gay prejudice: An application of personal construct theory. J Couns Psychol 2006;53(1):57-66.

320. Moradi B, van den Berg JJ, Epting FR. Threat and guilt aspects of internalized antilesbian and gay prejudice: An application of personal construct theory. Journal of Counseling Psychology 2009;56(1):119-131.

321. Moradi B, Wiseman MC, DeBlaere C, Goodman MB, Sarkees A, Brewster ME, et al. LGB of color and white individuals’ perceptions of heterosexist stigma, internalized homophobia, and outness: Comparisons of levels and links. The Counseling Psychologist 2010;38(3):397-424.

322. Moral-De-Rubia J, Martínez-Sulvarán J-O. Validación de la Escala de 10 ítems de Actitud hacia la Homosexualidad (EAH-10). Revista de Psicología Social 2012;27(2):183-197.

323. Morales Knight LF, Hope DA. Correlates of same-sex attractions and behaviors among self-identified heterosexual university students. Arch Sex Behav 2012;41(5):1199-208.

324. Morman MT, Schrodt P, Tornes MJ. Self-disclosure mediates the effects of gender orientation and homophobia on the relationship quality of male same-sex friendships. Journal of Social and Personal Relationships 2013;30(5):582-605.

325. Morrison MA. Psychological Health Correlates of Perceived Discrimination among Canadian Gay Men and Lesbian Women. Canadian Journal of Community Mental Health 2011;30(2):81-98.

326. Morrison MA, Morrison TG. Sexual orientation bias toward gay men and lesbian women: Modern homonegative attitudes and their association with discriminatory behavioral intentions. Journal of Applied Social Psychology 2011;41(11):2573-2599.

327. Morrison MA, Morrison TG, Franklin R. Modern and old-fashioned homonegativity among samples of Canadian and American university students. Journal of Cross-Cultural Psychology 2009;40(4):523-542.

328. Morrison TG, Bearden AG. The construction and validation of the homopositivity scale: an instrument measuring endorsement of positive stereotypes about gay men. J Homosex 2007;52(3-4):63-89.

329. Morrison TG, Harrington R, McDermott DT. Bi now, gay later: Implicit and explicit binegativity among Irish university students. Journal of Bisexuality 2010;10(3):211-232.

330. Morrison TG, Kenny P, Harrington A. Modern prejudice toward gay men and lesbian women: assessing the viability of a measure of modern homonegative attitudes within an Irish context. Genet Soc Gen Psychol Monogr 2005;131(3):219-50.

331. Morrison TG, Speakman C, Ryan TA. Irish university students’ support for the human rights of lesbian women and gay men. Journal of Homosexuality 2009;56(3):387-400.

332. Moskowitz DA, Rieger G, Roloff ME. Heterosexual attitudes toward same-sex marriage. J Homosex 2010;57(2):325-36.

333. Mudrey R, Medina-Adams A. Attitudes, perceptions, and knowledge of pre-service teachers regarding the educational isolation of sexual minority youth. J Homosex 2006;51(4):63-90.

334. Mulick PS, Wright LW, Jr. The Biphobia Scale a decade later: Reflections and additions. Journal of Bisexuality 2011;11(4):453-457.

335. Mullins MH. The relationship of practice beliefs and practice behaviors among social workers with lesbian and gay clients. Journal of Human Behavior in the Social Environment 2012;22(8):1050-1064.

336. Mustanski B, Greene GJ, Ryan D, Whitton SW. Feasibility, Acceptability, and Initial Efficacy of an Online Sexual Health Promotion Program for LGBT Youth: The Queer Sex Ed Intervention. J Sex Res 2014.

337. Nadler JT, Will K, Lowery MR, Smith K. Don't ask, don't tell and other LGB civil rights issues: Effects of terminology on public opinion. Journal of Gay & Lesbian Social Services: The Quarterly Journal of Community & Clinical Practice 2012;24(4):331-345.

338. Nagoshi JL, Adams KA, Terrell HK, Hill ED, Brzuzy S, Nagoshi CT. Gender differences in correlates of homophobia and transphobia. Sex Roles 2008;59(7-8):521-531.

339. Nakamura N, Zea MC. Experiences of homonegativity and sexual risk behaviour in a sample of Latino gay and bisexual men. Cult Health Sex 2010;12(1):73-85.

340. Negy C, Eisenman R. A comparison of African American and white college students' affective and attitudinal reactions to lesbian, gay, and bisexual individuals: an exploratory study. J Sex Res 2005;42(4):291-8.

341. Neilands TB, Steward WT, Choi KH. Assessment of stigma towards homosexuality in China: a study of men who have sex with men. Arch Sex Behav 2008;37(5):838-44.

342. Ngamake ST, Walch SE, Raveepatarakul J. Validation of the Coping With Discrimination Scale in Sexual Minorities. J Homosex 2013.

343. Nicol P, Chapman R, Watkins R, Young J, Shields L. Tertiary paediatric hospital health professionals' attitudes to lesbian, gay, bisexual and transgender parents seeking health care for their children. J Clin Nurs 2013;22(23-24):3396-405.

344. Nierman AJ, Thompson SC, Bryan A, Mahaffey AL. Gender role beliefs and attitudes toward lesbians and gay men in Chile and the U.S. Sex Roles 2007;57(1-2):61-67.

345. Norton AT, Herek GM. Heterosexuals' attitudes toward transgender people: Findings from a national probability sample of U.S. adults. Sex Roles 2013;68(11-12):738-753.

346. Nyamathi A, Branson C, Idemundia F, Reback C, Shoptaw S, Marfisee M, et al. Correlates of depressed mood among young stimulant-using homeless gay and bisexual men. Issues Ment Health Nurs 2012;33(10):641-9.

347. O'Brien KS, Caputi P, Minto R, Peoples G, Hooper C, Kell S, et al. Upward and downward physical appearance comparisons: development of scales and examination of predictive qualities. Body Image 2009;6(3):201-6.

348. O'Brien KS, Shovelton H, Latner JD. Homophobia in physical education and sport: the role of physical/sporting identity and attributes, authoritarian aggression, and social dominance orientation. Int J Psychol 2013;48(5):891-9.

349. O'Bryan M, Fishbein HD, Ritchey PN. Intergenerational transmission of prejudice, sex role stereotyping, and intolerance. Adolescence 2004;39(155):407-26.

350. Oksal A. Turkish family members' attitudes toward lesbians and gay men. Sex Roles 2008;58(7-8):514-525.

351. O'Leary A, Fisher HH, Purcell DW, Spikes PS, Gomez CA. Correlates of risk patterns and race/ethnicity among HIV-positive men who have sex with men. AIDS Behav 2007;11(5):706-15.

352. O'Leary A, Purcell DW, Remien RH, Fisher HE, Spikes PS. Characteristics of bisexually active men in the Seropositive Urban Mens' Study (SUMS). AIDS Care 2007;19(7):940-6.

353. Ortiz Hernandez L, Garcia Torres MI. [Internalized oppression and high-risk sexual practices among homosexual and bisexual males, Mexico]. Rev Saude Publica 2005;39(6):956-64.

354. Ortiz-Hernández L. Influencia de la opresión internalizada sobre la salud mental de bisexuales, lesbianas y homosexuales de la cuidad de México. Salud Mental 2005;28(4):49-65.

355. Oswald DL. 'Don't ask, don't tell': The influence of stigma concealing and perceived threat on perceivers' reactions to a gay target. Journal of Applied Social Psychology 2007;37(5):928-947.

356. Oswalt SB, Vargas TM. How safe is the playing field? Collegiate coaches' attitudes towards gay, lesbian, and bisexual individuals. Sport in Society 2013;16(1):120-132.

357. Oth VT, Lindner NM, Nosek BA. Do unto others: Effects of priming the golden rule on Buddhists and Christians attitudes toward gay people. Journal for the Scientific Study of Religion 2010;49(3):494-506.

358. Otis MD, Rostosky SS, Riggle EDB, Hamrin R. Stress and relationship quality in same-sex couples. Journal of Social & Personal Relationships 2006;23(1):81-99.

359. Pachankis JE, Goldfried MR, Ramrattan ME. Extension of the rejection sensitivity construct to the interpersonal functioning of gay men. J Consult Clin Psychol 2008;76(2):306-17.

360. Pachankis JE, Hatzenbuehler ML, Starks TJ. The influence of structural stigma and rejection sensitivity on young sexual minority men's daily tobacco and alcohol use. Soc Sci Med 2014;103:67-75.

361. Pacilli MG, Taurino A, Jost JT, van der Toorn J. System justification, right-wing conservatism, and internalized homophobia: Gay and lesbian attitudes toward same-sex parenting in Italy. Sex Roles 2011;65(7-8):580-595.

362. Papadaki V, Plotnikof K, Papadaki E. Social work students' attitudes towards lesbians and gay men: The case of the social work department in Crete, Greece. Social Work Education 2013;32(4):453-467.

363. Parnell MK, Lease SH, Green ML. Perceived career barriers for gay, lesbian, and bisexual individuals. Journal of Career Development 2012;39(3):248-268.

364. Parrott DJ. Aggression toward gay men as gender role enforcement: effects of male role norms, sexual prejudice, and masculine gender role stress. J Pers 2009;77(4):1137-66.

365. Parrott DJ, Peterson JL. What motivates hate crimes based on sexual orientation? Mediating effects of anger on antigay aggression. Aggress Behav 2008;34(3):306-18.

366. Parrott DJ, Peterson JL, Vincent W, Bakeman R. Correlates of anger in response to gay men: Effects of male gender role beliefs, sexual prejudice, and masculine gender role stress. Psychology of Men & Masculinity 2008;9(3):167-178.

367. Parrott DJ, Zeichner A. Effects of Sexual Prejudice and Anger on Physical Aggression Toward Gay and Heterosexual Men. Psychology of Men & Masculinity 2005;6(1):3-17.

368. Pelullo CP, Di Giuseppe G, Angelillo IF. Frequency of discrimination, harassment, and violence in lesbian, gay men, and bisexual in Italy. PLoS One 2013;8(8):e74446.

369. Perez-Testor C, Behar J, Davins M, Conde Sala JL, Castillo JA, Salamero M, et al. Teachers' attitudes and beliefs about homosexuality. Span J Psychol 2010;13(1):138-55.

370. Pettijohn TF, II, Walzer AS. Reducing racism, sexism, and homophobia in college students by completing a psychology of prejudice course. College Student Journal 2008;42(2, Pt A):459-468.

371. Pitpitan EV, Strathdee SA, Semple SJ, Wagner KD, Chavarin CV, Earnshaw VA, et al. Perceived Stigma of Purchasing Sex Among Latino and Non-Latino Male Clients of Female Sex Workers in Tijuana, Mexico. J Immigr Minor Health 2013.

372. Ploderl M, Sellmeier M, Fartacek C, Pichler EM, Fartacek R, Kralovec K. Explaining the Suicide Risk of Sexual Minority Individuals by Contrasting the Minority Stress Model with Suicide Models. Arch Sex Behav 2014.

373. Plumm KM, Terrance CA, Henderson VR, Ellingson H. Victim blame in a hate crime motivated by sexual orientation. J Homosex 2010;57(2):267-86.

374. Poteat VP. Peer group socialization of homophobic attitudes and behavior during adolescence. Child Dev 2007;78(6):1830-42.

375. Prati G. A social cognitive learning theory of homophobic aggression among adolescents. School Psychology Review 2012;41(4):413-428.

376. Prati G. Development and psychometric properties of the Homophobic Bullying Scale. Educational and Psychological Measurement 2012;72(4):649-664.

377. Prati G, Pietrantoni L, D'Augelli AR. Aspects of homophobia in Italian high schools: Students' attitudes and perceptions of school climate. Journal of Applied Social Psychology 2011;41(11):2600-2620.

378. Preston DB, D'Augelli AR, Kassab CD, Cain RE, Schulze FW, Starks MT. The influence of stigma on the sexual risk behavior of rural men who have sex with men. AIDS Educ Prev 2004;16(4):291-303.

379. Preston DB, D'Augelli AR, Kassab CD, Starks MT. The relationship of stigma to the sexual risk behavior of rural men who have sex with men. AIDS Educ Prev 2007;19(3):218-30.

380. Pyun T, Santos GM, Arreola S, Do T, Hebert P, Beck J, et al. Internalized Homophobia and Reduced HIV Testing Among Men Who Have Sex With Men in China. Asia Pac J Public Health 2014.

381. Radcliffe J, Doty N, Hawkins LA, Gaskins CS, Beidas R, Rudy BJ. Stigma and sexual health risk in HIV-positive African American young men who have sex with men. AIDS Patient Care STDS 2010;24(8):493-9.

382. Rainey S, Trusty J. Attitudes of master's-level counseling students toward gay men and lesbians. Counseling and Values 2007;52(1):12-24.

383. Raiz L, Saltzburg S. Developing awareness of the subtleties of heterosexism and homophobia among undergraduate, heterosexual social work majors. The Journal of Baccalaureate Social Work 2007;12(2):53-69.

384. Ramirez-Valles J, Fergus S, Reisen CA, Poppen PJ, Zea MC. Confronting stigma: community involvement and psychological well-being among HIV-positive Latino gay men. Hispanic Journal of Behavioral Sciences 2005;27(1):101-119.

385. Ramirez-Valles J, Kuhns LM, Campbell RT, Diaz RM. Social integration and health: community involvement, stigmatized identities, and sexual risk in Latino sexual minorities. J Health Soc Behav 2010;51(1):30-47.

386. Ramirez-Valles J, Molina Y, Dirkes J. Stigma towards PLWHA: the role of internalized homosexual stigma in Latino gay/bisexual male and transgender communities. AIDS Educ Prev 2013;25(3):179-89.

387. Ratcliff JJ, Lassiter GD, Markman KD, Snyder CJ. Gender differences in attitudes toward gay men and lesbians: the role of motivation to respond without prejudice. Pers Soc Psychol Bull 2006;32(10):1325-38.

388. Reid JA. Risk and resiliency factors influencing onset and adolescence-limited commercial sexual exploitation of disadvantaged girls. Crim Behav Ment Health 2014.

389. Reilly A, Rudd NA. Is Internalized Homonegativity Related to Body Image? Family and Consumer Sciences Research Journal 2006;35(1):58-73.

390. Reilly A, Rudd NA. Shopping behaviour among gay men: Issues of internalized homophobia and self-esteem. International Journal of Consumer Studies 2007;31(4):333-339.

391. Reisen CA, Brooks KD, Zea MC, Poppen PJ, Bianchi FT. Can additive measures add to an intersectional understanding? Experiences of gay and ethnic discrimination among HIV-positive Latino gay men. Cultur Divers Ethnic Minor Psychol 2013;19(2):208-17.

392. Rendina HJ, Golub SA, Grov C, Parsons JT. Stigma and sexual compulsivity in a community-based sample of HIV-positive gay and bisexual men. AIDS Behav 2012;16(3):741-50.

393. Riggle ED, Rostosky SS, Danner F. LGB identity and eudaimonic well being in midlife. J Homosex 2009;56(6):786-98.

394. Riggle ED, Rostosky SS, Horne SG. Psychological distress, well-being, and legal recognition in same-sex couple relationships. J Fam Psychol 2010;24(1):82-6.

395. Riggle EDB, Rostosky SS, Horne S. Does it matter where you live? Nondiscrimination laws and the experiences of LGB residents. Sexuality Research & Social Policy: A Journal of the NSRC 2010;7(3):168-175.

396. Rios K. Right-wing authoritarianism predicts prejudice against “homosexuals” but not “gay men and lesbians”. Journal of Experimental Social Psychology 2013;49(6):1177-1183.

397. Rivers I. Recollections of bullying at school and their long-term implications for lesbians, gay men, and bisexuals. Crisis 2004;25(4):169-75.

398. Robinson MA, Brewster ME. Motivations for fatherhood: Examining internalized heterosexism and gender-role conflict with childless gay and bisexual men. Psychology of Men & Masculinity 2014;15(1):49-59.

399. Rodríguez-Castro Y, Lameiras-Fernández M, Carrera-Fernández V, Vallejo-Medina P. Validación de la Escala de Homofobia Moderna en una muestra de adolescentes. Anales de Psicología 2013;29(2):523-533.

400. Rogers A, McRee N, Arntz DL. Using a college human sexuality course to combat homophobia. Sex Education 2009;9(3):211-225.

401. Rohner JC, Bjorklund F. Do self-presentation concerns moderate the relationship between implicit and explicit homonegativity measures? Scand J Psychol 2006;47(5):379-85.

402. Rondahl G, Innala S, Carlsson M. Nurses' attitudes towards lesbians and gay men. J Adv Nurs 2004;47(4):386-92.

403. Roper EA, Halloran E. Attitudes toward gay men and lesbians among heterosexual male and female student-athletes. Sex Roles 2007;57(11-12):919-928.

404. Rosenthal L, Levy SR, Moss I. Polyculturalism and openness about criticizing one’s culture: Implications for sexual prejudice. Group Processes & Intergroup Relations 2012;15(2):149-165.

405. Rosik CH. Ideological concerns in the operationalization of homophobia, part I: An analysis of Herek's ATLG-R scale. Journal of Psychology and Theology 2007;35(2):132-144.

406. Rosik CH, Dinges LJ, Saavedra N. Moral intuitions and attitudes toward gay men: Can moral psychology add to our understanding of homonegativity? Journal of Psychology and Theology 2013;41(4):315-326.

407. Rosik CH, Griffith LK, Cruz Z. Homophobia and conservative religion: toward a more nuanced understanding. Am J Orthopsychiatry 2007;77(1):10-9.

408. Ross MW, Berg RC, Schmidt AJ, Hospers HJ, Breveglieri M, Furegato M, et al. Internalised homonegativity predicts HIV-associated risk behavior in European men who have sex with men in a 38-country cross-sectional study: some public health implications of homophobia. BMJ Open 2013;3(2).

409. Ross MW, Kajubi P, Mandel JS, McFarland W, Raymond HF. Internalized homonegativity/homophobia is associated with HIV-risk behaviours among Ugandan gay and bisexual men. Int J STD AIDS 2013;24(5):409-13.

410. Ross MW, Rosser BR, Neumaier ER. The relationship of internalized homonegativity to unsafe sexual behavior in HIV-seropositive men who have sex with men. AIDS Educ Prev 2008;20(6):547-57.

411. Ross MW, Smolenski DJ, Kajubi P, Mandel JS, McFarland W, Raymond FH. Measurement of internalized homonegativity in gay and bisexual men in Uganda: Cross-cultural properties of the Internalized Homonegativity scale. Psychol Health Med 2010;15(2):159-65.

412. Ross MW, Timpson SC, Williams ML, Amos C, Bowen A. Stigma consciousness concerns related to drug use and sexuality in a sample of street-based male sex workers. International Journal of Sexual Health 2007;19(2):57-67.

413. Rosser BR, Bockting WO, Ross MW, Miner MH, Coleman E. The relationship between homosexuality, internalized homo-negativity, and mental health in men who have sex with men. J Homosex 2008;55(2):185-203.

414. Rottenbacher de Rojas JM. Conservadurismo político, homofobia y prejuicio hacia grupos transgénero en una muestra de estudiantes y egresados universitarios de Lima. Pensamiento Psicológico 2012;10(1):23-37.

415. Russell ST, Ryan C, Toomey RB, Diaz RM, Sanchez J. Lesbian, Gay, Bisexual, and Transgender Adolescent School Victimization: Implications for Young Adult Health and Adjustment. Journal of School Health 2011;81(5):223-230.

416. Rutledge SE, Siebert DC, Siebert C, Chonody J. Attitudes toward gays and lesbians: A latent class analysis of university students. Journal of Social Service Research 2011;38(1):18-28.

417. Rye BJ, Meaney GJ. Impact of a homonegativity awareness workshop on attitudes toward homosexuality. J Homosex 2009;56(1):31-55.

418. Rye BJ, Meaney GJ. Measuring homonegativity: a psychometric analysis. Canadian Journal of Behavioural Science 2010;42(3):158-167.

419. Sanabria S. Religious Orientation and Prejudice: Predictors of Homoprejudice. Journal of LGBT Issues in Counseling 2012;6(3):183-201.

420. Sanchez FJ, Westefeld JS, Liu WM, Vilain E. Masculine Gender Role Conflict and Negative Feelings about Being Gay. Prof Psychol Res Pr 2010;41(2):104-111.

421. Sanchez T, Finlayson T, Murrill C, Guilin V, Dean L. Risk behaviors and psychosocial stressors in the new york city house ball community: a comparison of men and transgender women who have sex with men. AIDS Behav 2010;14(2):351-8.

422. Saraç L. The relationships between homophobic attitudes and religiosity among Turkish physical education teacher majors. Physical Education and Sport Pedagogy 2012;17(3):277-287.

423. Saraç L. Attitudes of future physical education teachers in Turkey toward lesbians and gay men. Psychological Reports 2012;111(3):765-775.

424. Sartore ML, Cunningham GB. Gender, sexual prejudice and sport participation: Implications for sexual minorities. Sex Roles 2009;60(1-2):100-113.

425. Satcher J, Schumacker R. Predictors of modern homonegativity among professional counselors. Journal of LGBT Issues in Counseling 2009;3(1):21-36.

426. Savage TA, Prout HT, Chard KM. School psychology and issues of sexual orientation: Attitudes, beliefs, and knowledge. Psychology in the Schools 2004;41(2):201-210.

427. Schiappa E, Gregg PB, Hewes DE. Can one TV show make a difference? Will & Grace and the Parasocial Contact Hypothesis. J Homosex 2006;51(4):15-37.

428. Schmidt CK, Miles JR, Welsh AC. Perceived discrimination and social support: The influences on career development and college adjustment of LGBT college students. Journal of Career Development 2011;38(4):293-309.

429. Schneider MS, Dimito A. Factors influencing the career and academic choices of lesbian, gay, bisexual, and transgender people. J Homosex 2010;57(10):1355-69.

430. Schrimshaw EW, Siegel K, Downing MJ, Parsons JT. Disclosure and concealment of sexual orientation and the mental health of non-gay-identified, behaviorally bisexual men. J Consult Clin Psychol 2013;81(1):141-53.

431. Schulte LJ, Battle J. The Relative Importance of Ethnicity and Religion in Predicting Attitudes Towards Gays and Lesbians. Journal of Homosexuality 2004;47(2):127-141.

432. Scott HM, Pollack L, Rebchook GM, Huebner DM, Peterson J, Kegeles SM. Peer Social Support is Associated with Recent HIV Testing Among Young Black Men Who Have Sex with Men. AIDS Behav 2013.

433. Shen MJ, Yelderman LA, Haggard MC, Rowatt WC. Disentangling the belief in God and cognitive rigidity/flexibility components of religiosity to predict racial and value-violating prejudice: A Post-Critical Belief Scale analysis. Personality and Individual Differences 2013;54(3):389-395.

434. Sherry A. Internalized homophobia and adult attachment: Implications for clinical practice. Psychotherapy (Chic) 2007;44(2):219-25.

435. Sherry A, Adelman A, Whilde MR, Quick D. Competing selves: Negotiating the intersection of spiritual and sexual identities. Professional Psychology: Research and Practice 2010;41(2):112-119.

436. Shilo G, Mor Z. The impact of minority stressors on the mental and physical health of young gays, lesbians and bisexuals. Journal of Sexual Medicine 2012;9:332-333.

437. Shilo G, Savaya R. Mental health of lesbian, gay, and bisexual youth and young adults: Differential effects of age, gender, religiosity, and sexual orientation. Journal of Research on Adolescence 2012;22(2):310-325.

438. Shoptaw S, Weiss RE, Munjas B, Hucks-Ortiz C, Young SD, Larkins S, et al. Homonegativity, substance use, sexual risk behaviors, and HIV status in poor and ethnic men who have sex with men in Los Angeles. J Urban Health 2009;86 Suppl 1:77-92.

439. Siebert DC, Chonody J, Rutledge SE, Killian M. The Index of Attitudes toward Homosexuals 30 years later: a psychometric study. Research on Social Work Practice 2009;19(2):214-220.

440. Smith B, Horne S. Gay, lesbian, bisexual and transgendered (GLBT) experiences with Earth-spirited faith. J Homosex 2007;52(3-4):235-48.

441. Smith DM, Mathews WC. Physician's attitude toward homosexuality and HIV: Survey of a California Medical Society Revisited (PATHH-II). Journal of Homosexuality 2007;52(3-4):1-9.

442. Smith NG, Ingram KM. Workplace Heterosexism and Adjustment Among Lesbian, Gay, and Bisexual Individuals: The Role of Unsupportive Social Interactions. Journal of Counseling Psychology 2004;51(1):57-67.

443. Smith S, Zanotti, DC, Axelton, AM, & Saucier, DA. Individuals' Beliefs about the Etiology of Same-Sex Sexual Orientation. Journal of Homosexuality 2011;58:1110-1131.

444. Smith WP. Exploring dimensions of racism, homophobia, and social network as concomitant predictors of condom use in a sample of Black MSM. Journal of Gay & Lesbian Social Services: The Quarterly Journal of Community & Clinical Practice 2012;24(4):417-445.

445. Smith WP. Exploring the validity and statistical utility of a racism scale among Black men who have sex with men: a pilot study. Am J Mens Health 2013;7(5):362-73.

446. Smolenski DJ, Diamond PM, Ross MW, Rosser BR. Revision, criterion validity, and multigroup assessment of the reactions to homosexuality scale. J Pers Assess 2010;92(6):568-76.

447. Smolenski DJ, Ross MW, Risser JM, Rosser BR. Sexual compulsivity and high-risk sex among Latino men: the role of internalized homonegativity and gay organizations. AIDS Care 2009;21(1):42-9.

448. Smolenski DJ, Stigler MH, Ross MW, Rosser BR. Direct and indirect effects between internalized homonegativity and high-risk sex. Arch Sex Behav 2011;40(4):785-92.

449. Snively CA, Kreuger L, Stretch JJ, Watt JW, Chadha J. Understanding homophobia: preparing for practice realities in urban and rural settings. J Gay Lesbian Soc Serv 2004;17(1):59-81.

450. Span SA. Addressing university students’ anti-gay bias: An extension of the contact hypothesis. American Journal of Sexuality Education 2011;6(2):192-205.

451. Span SA, Derby PL. Depressive symptoms moderate the relation between internalized homophobia and drinking habits. Journal of Gay & Lesbian Social Services: Issues in Practice, Policy & Research 2009;21(1):1-12.

452. Starks TJ, Payton G, Golub SA, Weinberger CL, Parsons JT. Contextualizing Condom Use: Intimacy Interference, Stigma, and Unprotected Sex. J Health Psychol 2013.

453. Starks TJ, Rendina HJ, Breslow AS, Parsons JT, Golub SA. The psychological cost of anticipating HIV stigma for HIV-negative gay and bisexual men. AIDS Behav 2013;17(8):2732-41.

454. Steffens MC. Implicit and explicit attitudes towards lesbians and gay men. J Homosex 2005;49(2):39-66.

455. Steffens MC, Wagner C. Attitudes toward lesbians, gay men, bisexual women, and bisexual men in Germany. J Sex Res 2004;41(2):137-49.

456. Stoever CJ, Morera OF. A confirmatory factor analysis of the Attitude Toward Lesbians and Gay Men (ATLG) measure. Journal of Homosexuality 2007;52(3-4):189-209.

457. Stotzer RL, Shih M. The Relationship Between Masculinity and Sexual Prejudice in Factors Associated With Violence Against Gay Men. Psychology of Men & Masculinity 2012;13(2):136-142.

458. Sümer ZH. Effects of gender and sex-role orientation on sexual attitudes among Turkish university students. Social Behavior and Personality 2013;41(6):995-1008.

459. Swank E, Raiz L. Explaining comfort with homosexuality among social work students: The impact of demographic, contextual, and attitudinal factors. Journal of Social Work Education 2007;43(2):257-279.

460. Szymanski D, Hilton A. Fear of Intimacy as a Mediator of the Internalized Heterosexism-Relationship Quality Link Among Men in Same-Sex Relationships. Contemporary Family Therapy: An International Journal 2013;35(4):760-772.

461. Szymanski DM. Examining potential moderators of the link between heterosexist events and gay and bisexual men's psychological distress. Journal of Counseling Psychology 2009;56(1):142-151.

462. Szymanski DM, Carr ER. The roles of gender role conflict and internalized heterosexism in gay and bisexual men's psychological distress: Testing two mediation models. Psychology of Men & Masculinity 2008;9(1):40-54.

463. Szymanski DM, Gupta A. Examining the relationships between multiple oppressions and Asian American sexual minority persons’ psychological distress. Journal of Gay & Lesbian Social Services: Issues in Practice, Policy & Research 2009;21(2-3):267-281.

464. Szymanski DM, Ikizler AS. Internalized heterosexism as a mediator in the relationship between gender role conflict, heterosexist discrimination, and depression among sexual minority men. Psychology of Men & Masculinity 2013;14(2):211-219.

465. Szymanski DM, Sung MR. Minority stress and psychological distress among Asian American sexual minority persons. The Counseling Psychologist 2010;38(6):848-872.

466. Szymanski DM, Sung MR. Asian Cultural Values, Internalized Heterosexism, and Sexual Orientation Disclosure Among Asian American Sexual Minority Persons. Journal of LGBT Issues in Counseling 2013;7(3):257-273.

467. Talley AE, Bettencourt BA. The moderator roles of coping style and identity disclosure in the relationship between perceived sexual stigma and psychological distress. Journal of Applied Social Psychology 2011;41(12):2883-2903.

468. Tebbe EN, Moradi B. Anti-transgender prejudice: a structural equation model of associated constructs. J Couns Psychol 2012;59(2):251-61.

469. Teney C, Subramanian SV. Attitudes toward homosexuals among youth in multiethnic Brussels. Cross-Cultural Research: The Journal of Comparative Social Science 2010;44(2):151-173.

470. Theodore JL, Shidlo A, Zemon V, Foley FW, Dorfman D, Dahlman KL, et al. Psychometrics of an internalized homophobia instrument for men. J Homosex 2013;60(4):558-74.

471. Thoma BC, Huebner DM. Health consequences of racist and antigay discrimination for multiple minority adolescents. Cultur Divers Ethnic Minor Psychol 2013;19(4):404-13.

472. Thomas B, Mimiaga MJ, Mayer KH, Perry NS, Swaminathan S, Safren SA. The influence of stigma on HIV risk behavior among men who have sex with men in Chennai, India. AIDS Care 2012;24(11):1401-6.

473. Toro-Alfonso J, Varas-Díaz N. Los otros: Prejuicio y distancia social hacia hombres gay y lesbianas en una muestra de estudiantes de nivel universitario. International Journal of Clinical and Health Psychology 2004;4(3):537-551.

474. Torres ARR, de Faria MRGV. Creencia en un mundo justo y prejuicios: El caso de los homosexuales con VIH/SIDA. Revista Interamericana de Psicología 2008;42(3):570-579.

475. Torres H, Delonga K, Lee S, Gladstone KA, Barrad A, Huckaby S, et al. Socio-Contextual Factors: Moving Beyond Individual Determinants of Sexual Risk Behavior among Gay and Bisexual Adolescent Males. J LGBT Youth 2013;10(3).

476. Tozer EE, Hayes JA. Why Do individuals seek conversion therapy?: The role of religiosity, internalized homonegativity, and identity development. The Counseling Psychologist 2004;32(5):716-740.

477. Trevino KM, Desai K, Lauricella S, Pargament KI, Mahoney A. Perceptions of lesbian and gay (LG) individuals as desecrators of Christianity as predictors of anti-LG attitudes. J Homosex 2012;59(4):535-63.

478. Tsang J-A, Rowatt WC. The relationship between religious orientation, right-wing authoritarianism, and implicit sexual prejudice. International Journal for the Psychology of Religion 2007;17(2):99-120.

479. Tu J, Lee TT. The Effects of Media Usage and Interpersonal Contacts on the Stereotyping of Lesbians and Gay Men in China. J Homosex 2013.

480. Tucker A, Liht J, de Swardt G, Jobson G, Rebe K, McIntyre J, et al. Homophobic stigma, depression, self-efficacy and unprotected anal intercourse for peri-urban township men who have sex with men in Cape Town, South Africa: a cross-sectional association model. AIDS Care 2013.

481. Tucker-Seeley RD, Blow AJ, Matsuo H, Taylor-Moore R. Behavioral escape avoidance coping in African-American men who have sex with men. Journal of Gay & Lesbian Social Services: The Quarterly Journal of Community & Clinical Practice 2010;22(3):250-268.

482. van den Akker H, van der Ploeg R, Scheepers P. Disapproval of homosexuality: Comparative research on individual and national determinants of disapproval of homosexuality in 20 European countries. International Journal of Public Opinion Research 2013;25(1):64-86.

483. van der Elst EM, Smith AD, Gichuru E, Wahome E, Musyoki H, Muraguri N, et al. Men who have sex with men sensitivity training reduces homoprejudice and increases knowledge among Kenyan healthcare providers in coastal Kenya. J Int AIDS Soc 2013;16 Suppl 3:18748.

484. Vega MY, Spieldenner AR, DeLeon D, Nieto BX, Stroman CA. SOMOS: evaluation of an HIV prevention intervention for Latino gay men. Health Educ Res 2011;26(3):407-18.

485. Velez BL, Moradi B. Workplace support, discrimination, and person–organization fit: Tests of the theory of work adjustment with LGB individuals. Journal of Counseling Psychology 2012;59(3):399-407.

486. Velez BL, Moradi B, Brewster ME. Testing the tenets of minority stress theory in workplace contexts. Journal of Counseling Psychology 2013;60(4):532-542.

487. Ventura LA, Lambert EG, Bryant M, Pasupuleti S. Differences in Attitudes Toward Gays and Lesbians Among Criminal Justice and Non-Criminal Justice Majors. American Journal of Criminal Justice 2004;28(2):165-180.

488. Verduzco IL, Díaz-Loving R. Medición de la homofobia en México: Desarrollo y validación. Revista Iberoamericana de Diagnóstico y Evaluación/E Avaliação Psicológica 2010;30(2):105-124.

489. Verweij KJ, Shekar SN, Zietsch BP, Eaves LJ, Bailey JM, Boomsma DI, et al. Genetic and environmental influences on individual differences in attitudes toward homosexuality: an Australian twin study. Behav Genet 2008;38(3):257-65.

490. Vicario BA, Liddle BJ, Luzzo DA. The role of values in understanding attitudes toward lesbians and gay men. J Homosex 2005;49(1):145-59.

491. Vincent W, Parrott DJ, Peterson JL. Combined effects of masculine gender‐role stress and sexual prejudice on anger and aggression toward gay men. Journal of Applied Social Psychology 2011;41(5):1237-1257.

492. Vincent W, Parrott DJ, Peterson JL. Effects of Traditional Gender Role Norms and Religious Fundamentalism on Self-Identified Heterosexual Men's Attitudes, Anger, and Aggression Toward Gay Men and Lesbians. Psychol Men Masc 2011;12(4):383-400.

493. Vincent W, Peterson JL, Parrott DJ. Differences in African American and White Women's Attitudes Toward Lesbians and Gay Men. Sex Roles 2009;61(9-10):599-606.

494. Vu L, Tun W, Sheehy M, Nel D. Levels and correlates of internalized homophobia among men who have sex with men in Pretoria, South Africa. AIDS Behav 2012;16(3):717-23.

495. Walch SE, Ngamake ST, Francisco J, Stitt RL, Shingler KA. The attitudes toward transgendered individuals scale: psychometric properties. Arch Sex Behav 2012;41(5):1283-91.

496. Walch SE, Orlosky PM, Sinkkanen KA, Stevens HR. Demographic and social factors associated with homophobia and fear of AIDS in a community sample. J Homosex 2010;57(2):310-24.

497. Walch SE, Sinkkanen KA, Swain EM, Francisco J, Breaux CA, Sjoberg MD. Using intergroup contact theory to reduce stigma against transgender individuals: Impact of a transgender speaker panel presentation. Journal of Applied Social Psychology 2012;42(10):2583-2605.

498. Walker JJ, Longmire-Avital B. The impact of religious faith and internalized homonegativity on resiliency for black lesbian, gay, and bisexual emerging adults. Dev Psychol 2013;49(9):1723-31.

499. Wallenberg J, Anspach A, Leon AM. Student degree program and attitudes toward gays and lesbians in the military: is there a connection? J Homosex 2011;58(4):476-96.

500. Walls NE. Toward a multidimensional understanding of heterosexism: the changing nature of prejudice. J Homosex 2008;55(1):20-70.

501. Warriner K, Nagoshi CT, Nagoshi JL. Correlates of homophobia, transphobia, and internalized homophobia in gay or lesbian and heterosexual samples. J Homosex 2013;60(9):1297-314.

502. Weber GN. Using to numb the pain: Substance use and abuse among lesbian, gay and bisexual individuals. Journal of Mental Health Counseling 2008;30(1):31-48.

503. Webster RJ, Saucier DA. The effects of death reminders on sex differences in prejudice toward gay men and lesbians. J Homosex 2011;58(3):402-26.

504. Weiss BJ, Hope DA. A preliminary investigation of worry content in sexual minorities. J Anxiety Disord 2011;25(2):244-50.

505. West K, Cowell NM. Predictors of Prejudice Against Lesbians and Gay Men in Jamaica. J Sex Res 2014.

506. West K, Hewstone M. Culture and contact in the promotion and reduction of anti-gay prejudice: evidence from Jamaica and Britain. J Homosex 2012;59(1):44-66.

507. White D, Stephenson R. Identity formation, outness, and sexual risk among gay and bisexual men. Am J Mens Health 2014;8(2):98-109.

508. Whitley BE, Jr., Childs CE, Collins JB. Differences in Black and White American college students’ attitudes toward lesbians and gay men. Sex Roles 2011;64(5-6):299-310.

509. Wight RG, LeBlanc AJ, de Vries B, Detels R. Stress and mental health among midlife and older gay-identified men. Am J Public Health 2012;102(3):503-10.

510. Wiley TR, Bottoms BL. Attitudinal and individual differences influence perceptions of mock child sexual assault cases involving gay defendants. J Homosex 2013;60(5):734-49.

511. Wilkerson JM, Smolenski DJ, Brady SS, Rosser BR. Religiosity, Internalized Homonegativity, and Outness in Christian Men Who Have Sex with Men. Sex Relation Ther 2012;27(2):122-132.

512. Wilkinson WW. Religiosity, Authoritarianism, and Homophobia: A Multidimensional Approach. International Journal for the Psychology of Religion 2004;14(1):55-67.

513. Willoughby BLB, Hill DB, Gonzalez CA, Lacorazza A, Macapagal RA, Barton ME, et al. Who Hates Gender Outlaws? A Multisite and Multinational Evaluation of the Genderism and Transphobia Scale. International Journal of Transgenderism 2010;12(4):254-271.

514. Windsor LC, Benoit E, Ream GL, Forenza B. The provider perception inventory: psychometrics of a scale designed to measure provider stigma about HIV, substance abuse, and MSM behavior. AIDS Care 2013;25(5):586-91.

515. Winter S, Chalungsooth P, Teh YK, Rojanalert N, Maneerat K, Wong YW, et al. Transpeople, transprejudice and pathologization: a seven-country factor analytic study. International Journal of Sexual Health 2009;21(2):96-118.

516. Winter S, Webster B, Cheung PKE. Measuring Hong Kong undergraduate students' attitudes towards transpeople. Sex Roles 2008;59(9-10):670-683.

517. Wohl AR, Galvan FH, Carlos JA, Myers HF, Garland W, Witt MD, et al. A comparison of MSM stigma, HIV stigma and depression in HIV-positive Latino and African American men who have sex with men (MSM). AIDS Behav 2013;17(4):1454-64.

518. Wohl AR, Galvan FH, Myers HF, Garland W, George S, Witt M, et al. Do social support, stress, disclosure and stigma influence retention in HIV care for Latino and African American men who have sex with men and women? AIDS Behav 2011;15(6):1098-110.

519. Wolff JR, Himes HL, Kwon EM, Bollinger RA. Evangelical Christian College Students and Attitudes Toward Gay Rights: A California University Sample. Journal of LGBT Youth 2012;9(3):200-224.

520. Wong CF, Schrager SM, Holloway IW, Meyer IH, Kipke MD. Minority stress experiences and psychological well-being: the impact of support from and connection to social networks within the Los Angeles House and Ball communities. Prev Sci 2014;15(1):44-55.

521. Wong CF, Weiss G, Ayala G, Kipke MD. Harassment, discrimination, violence, and illicit drug use among young men who have sex with men. AIDS Educ Prev 2010;22(4):286-98.

522. Wong CY, Tang CS. Sexual practices and psychosocial correlates of current condom use among Chinese gay men in Hong Kong. Arch Sex Behav 2004;33(2):159-67.

523. Woodford MR, Atteberry B, Derr M, Howell M. Endorsement for Civil Rights for Lesbian, Gay, Bisexual, and Transgender People Among Heterosexual College Students: Informing Socially Just Policy Advocacy. Journal of Community Practice 2013;21(3):203-227.

524. Worthen MGF. College Student Experiences with an LGBTQ Ally Training Program: A Mixed Methods Study at a University in the Southern United States. Journal of LGBT Youth 2011;8(4):332-377.

525. Worthen MGF. Heterosexual college student sexual experiences, feminist identity, and attitudes toward LGBT individuals. Journal of LGBT Youth 2012;9(2):77-113.

526. Worthington RL, Dillon FR, Becker-Schutte AM. Development, Reliability, and Validity of the Lesbian, Gay, and Bisexual Knowledge and Attitudes Scale for Heterosexuals (LGB-KASH). Journal of Counseling Psychology 2005;52(1):104-118.

527. Wright AJ, Wegner RT. Homonegative Microaggressions and Their Impact on LGB Individuals: A Measure Validity Study. Journal of LGBT Issues in Counseling 2012;6(1):34-54.

528. Wright PJ, Bae S. Pornography consumption and attitudes toward homosexuality: A national longitudinal study. Human Communication Research 2013;39(4):492-513.

529. Wu J, Kwok DK. Psychometric properties of Attitudes Towards Lesbians and Gay Men Scale with Chinese university students. Psychological Reports 2012;110(2):521-526.

530. Wyer NA. Salient egalitarian norms moderate activation of out-group approach and avoidance. Group Processes & Intergroup Relations 2010;13(2):151-165.

531. Yen CF, Pan SM, Hou SY, Liu HC, Wu SJ, Yang WC, et al. Attitudes toward gay men and lesbians and related factors among nurses in Southern Taiwan. Public Health 2007;121(1):73-9.

532. Yi H, Sandfort TG, Shidlo A. Effects of disengagement coping with HIV risk on unprotected sex among HIV-negative gay men in New York City. Health Psychol 2010;29(2):205-14.

533. Yi H, Shidlo A, Sandfort T. Assessing maladaptive responses to the stress of being at risk of HIV Infection among HIV-negative gay men in New York City. J Sex Res 2011;48(1):62-73.

534. Yost MR, Gilmore S. Assessing LGBTQ campus climate and creating change. J Homosex 2011;58(9):1330-54.

535. Yost MR, Thomas GD. Gender and binegativity: men's and women's attitudes toward male and female bisexuals. Arch Sex Behav 2012;41(3):691-702.

536. Young SD, Shoptaw S, Weiss RE, Munjas B, Gorbach PM. Predictors of unrecognized HIV infection among poor and ethnic men who have sex with men in Los Angeles. AIDS Behav 2011;15(3):643-9.

537. Yu Y, Xiao S, Xiang Y. Application and testing the reliability and validity of a modified version of Herek's Attitudes Toward Lesbians and Gay Men Scale in China. Journal of Homosexuality 2011;58(2):263-274.

538. Zamboni BD, Robinson BBE, Bockting WO. HIV status and coming out among African American gay and bisexual men. Journal of Bisexuality 2011;11(1):75-85.

539. Zeichner A, Reidy DE. Are homophobic men attracted to or repulsed by homosexual men? Effects of gay male erotica on anger, fear, happiness, and disgust. Psychology of Men & Masculinity 2009;10(3):231-236.

540. Zhang C, Li X, Hong Y, Su S, Zhou Y. Relationship between female sex workers and gatekeeper: The impact on female sex worker's mental health in China. Psychol Health Med 2013.

541. Zivony A, Lobel T. The Invisible Stereotypes of Bisexual Men. Arch Sex Behav 2014.
